# Plant community controls on short‐term ecosystem nitrogen retention

**DOI:** 10.1111/nph.13832

**Published:** 2016-01-08

**Authors:** Franciska T. de Vries, Richard D. Bardgett

**Affiliations:** ^1^ Faculty of Life SciencesThe University of ManchesterOxford RoadManchesterM13 9PTUK

**Keywords:** competition, functional group, functional traits, leaf traits, nitrogen enrichment, plant–soil interactions, root traits, soil microbial community

## Abstract

- Retention of nitrogen (N) is a critical ecosystem function, especially in the face of widespread anthropogenic N enrichment; however, our understanding of the mechanisms involved is limited. Here, we tested under glasshouse conditions how plant community attributes, including variations in the dominance, diversity and range of plant functional traits, influence N uptake and retention in temperate grassland.
- We added a pulse of ^15^N to grassland plant communities assembled to represent a range of community‐weighted mean plant traits, trait functional diversity and divergence, and species richness, and measured plant and microbial uptake of ^15^N, and leaching losses of ^15^N, as a short‐term test of N retention in the plant–soil system.
- Root biomass, herb abundance and dominant plant traits were the main determinants of N retention in the plant–soil system: greater root biomass and herb abundance, and lower root tissue density, increased plant ^15^N uptake, while higher specific leaf area and root tissue density increased microbial ^15^N uptake.
- Our results provide novel, mechanistic insight into the short‐term fate of N in the plant–soil system, and show that dominant plant traits, rather than trait functional diversity, control the fate of added N in the plant–soil system.

## Introduction

Humans have irreversibly changed the global nitrogen (N) cycle by doubling the amount of reactive N in the biosphere, which has led to increased greenhouse gas emissions and nutrient enrichment of aquatic and terrestrial ecosystems (Galloway *et al*., 2008; Schlesinger, 2009). Chronic N enrichment can indirectly affect ecosystem functioning by reducing plant community diversity and encouraging dominance of fast‐growing, highly productive species (Stevens *et al*., 2004; Klumpp & Soussana, 2009), but also through its negative effects on mycorrhizal fungi (Bradley *et al*., 2006), which promote plant species coexistence and diversity (Van der Heijden *et al*., 1998; Wagg *et al*., 2011a,b). Ecosystem N retention is a critical ecosystem function, especially in the light of increased N loads, given that plants and soils together can retain significant amounts of N, thereby preventing it from being released to the surrounding environment. While it is well known that abiotic factors such as pH, soil texture and soil organic matter content affect ecosystem N retention, much less is known about the role of biotic factors, including interactions between plants and soil microbes (De Vries & Bardgett, 2012). This represents a significant gap in understanding, given growing evidence that plant–microbial linkages are likely to play a crucial role in determining the balance between N retained in plant and soils and the amount of N lost as gases or leachates to the surrounding environment (Suding *et al*., 2008).

Many studies have shown a positive effect of plant species richness on ecosystem N retention, which has been attributed to niche complementarity and overyielding, resulting in greater root uptake of available N (Hooper & Vitousek, 1998; Scherer‐Lorenzen *et al*., 2003; De Deyn *et al*., 2009; Bingham & Biondini, 2011), but also to the increased chance of diverse mixtures including a highly influential species (Mulder *et al*., 2002). The number and identity of functional groups present in a plant community can also affect N leaching. For example, legumes can increase N leaching through their positive effect on soil N availability (Scherer‐Lorenzen *et al*., 2003), while grasses can decrease N leaching, presumably through their dense root systems (Phoenix *et al*., 2008). However, it has also been found in experimental mesocosms that individual legume and forb species can decrease N leaching (De Deyn *et al*., 2009). To better understand these observed diversity, functional group and individual species effects on N leaching and other ecosystem functions, ecologists are increasingly turning to trait‐based approaches (Lavorel & Garnier, 2002; Diaz *et al*., 2007; De Deyn *et al*., 2008; Lavorel *et al*., 2013). These trait‐based explanations of plant community effects on ecosystem functioning make use of the leaf economics spectrum, which links leaf traits to plant resource uptake strategies and subsequent growth rates. Here, high specific leaf area (SLA) and high leaf N content (leaf N) are traits directly involved in photosynthesis and are representative of species with exploitative growth strategies, while high leaf dry matter content (LDMC) is involved in defence and longevity of above‐ground plant parts and linked to resource‐conservative growth strategies (Diaz *et al*., 2004; Wright *et al*., 2004). Recently, the focus of these trait‐based approaches has shifted below ground, although root traits are not as easily characterized into conservative or exploitative strategies as leaf traits (Mommer & Weemstra, 2012; Bardgett *et al*., 2014).

Plant species richness effects on primary productivity (Naeem, 2002), ecosystem carbon (C) fluxes (Milcu *et al*., 2014) and soil faunal community composition (Milcu *et al*., 2013) have been attributed to the diversity and divergence of functional traits, rather than species diversity *per se*. However, as yet, no studies have explicitly tested whether functional traits underlie plant species richness effects on processes of N cycling, although studies have linked plant traits related to N acquisition to microbial communities and their activities in monocultures. For example, exploitative plant traits, such as high SLA and leaf N content, have been linked to high soil N availability (Orwin *et al*., 2010), but also to greater plant N uptake (Grassein *et al*., 2015). Also, in pot experiments, plant N‐use efficiency (specific root N uptake) was found to reduce the abundance of nitrate‐reducing bacteria (Moreau *et al*., 2015), and root traits were shown to be stronger controls on potential rates of denitrification and nitrification than leaf traits (Cantarel *et al*., 2015). In addition, a number of field studies have explained N cycling processes using trait‐based approaches. A forest study demonstrated that dominance of exploitative plant traits, measured as community‐weighted means (CWMs), is linked to high rates of soil N cycling, and potentially high N losses through nitrification and denitrification (Laughlin, 2011). By contrast, grasslands dominated by conservative plant species can have greater ecosystem N retention, both in the field and in laboratory experiments (Suding *et al*., 2008; De Vries *et al*., 2012a, 2015; Grigulis *et al*., 2013). Thus, there is a gap in understanding between individual species and observational plant community effects on N cycling, namely how different components of plant functional diversity mechanistically affect ecosystem N retention or loss.

Although plant community effects on N retention can act directly, through root uptake and subsequent transport to above‐ground plant parts (De Vries *et al*., 2012a), short‐term ecosystem N retention mainly depends on N uptake by soil microbes (Zogg *et al*., 2000; De Vries *et al*., 2012a). Especially over short timescales (h to d), microbes are better competitors for N than plant roots and take up most of the soil available N, which can become available for plant uptake after subsequent remineralization (Kaye & Hart, 1997; Bardgett *et al*., 2003; De Vries *et al*., 2012a). Previous studies have suggested that competition for N is fiercer in plant communities dominated by conservative plant traits, and that this results in greater microbial immobilization of N (Harrison *et al*., 2008) and N retention, and lower N loss (De Vries & Bardgett, 2012) than in communities dominated by exploitative traits. However, evidence for this is inconsistent; a few field‐based studies have shown that grasslands dominated by conservative plant traits have smaller N leaching loss (De Vries *et al*., 2012a; Grigulis *et al*., 2013) through greater N immobilization by fungal‐dominated microbial communities (De Vries *et al*., 2012a), while others did not find evidence that plant growth strategies affected microbial N uptake in a glasshouse experiment (Harrison *et al*., 2008). In addition, a recent glasshouse experiment found that nitrate‐reducing bacteria were reduced under plants with high N‐uptake rates, but plant N uptake was not explained by plant traits (summarized in a nitrophily index, Moreau *et al*. (2015)). Thus, although it is well established that plant functional traits can affect plant N uptake and microbe‐mediated processes of N cycling, it is not clear how they influence microbial immobilization of N in soil or how they impact N retention, or the total amount of N retained in plants, microbes and soil.

Here, we used a unique experimental design involving a range of constructed plant communities to experimentally test how plant community attributes affect ecosystem N retention. We focused on dominant leaf traits, trait functional diversity and divergence, and used temperate grassland as a model system. We aimed to contrast the mass‐ratio hypothesis, which states that dominant plant species control ecosystem processes (Grime, 1998), and the functional diversity hypothesis (Johnson *et al*., 1996; Diaz *et al*., 2007), which states that trait functional diversity, rather than species diversity or dominant plant species *per se*, controls ecosystem processes. Thus, we compared two specific hypotheses on how plant community composition controls N retention: dominance of conservative plant traits increases N retention through greater plant uptake and through promoting microbial immobilization, thus increasing N retention and reducing N leaching; and trait functional diversity and trait functional divergence enhance N retention through greater above‐ground and below‐ground N uptake through niche complementarity and overyielding.

## Materials and Methods

### Experimental setup

We collected soil from mesotrophic grassland at Lancaster University's Hazelrigg Field Station in northern England (54°1′N, 2°46′W, 94 m above sea level). The soil used was a silt loam of the Brickfield 2 association, %N 0.19, %C 2.35, pH 4.75. Soil was sieved and homogenized (4 mm mesh size) and 2.5 kg field‐moist soil was packed in 3 l (19 cm diameter, 15 cm depth) pots.

We constructed grassland plant communities representing a range of CWM trait, functional diversity and functional divergence values using a species pool of 24 grassland species. Twelve grasses and 12 herb species representative of mesotrophic grassland were selected and ranked according to their SLA (from Grime *et al*., 2007; Table 1). We used SLA because this leaf trait is strongly correlated to leaf N, on both a local and a global scale, and these two traits have a strong physiological link with leaf photosynthesis (Wright *et al*., 2004). Moreover, both SLA and leaf N have previously been linked to soil microbial community composition and carbon (C) and N cycling in grasslands (De Vries *et al*., 2012b; Garcia‐Palacios *et al*., 2013; Grigulis *et al*., 2013; Milcu *et al*., 2014). Ranking the 24 grassland species resulted in three categories for both grasses and herbs: exploitative grasses and herbs with high SLA (category 1); conservative grasses and herbs with low SLA (category 3); and a group of grasses and herbs with intermediate SLA (category 2; Table 1). These trait categories were used to construct plant communities.

**Table 1:** The 24 grassland species (and abbreviations) that made up the species pool used to construct plant communities, with their specific leaf area (SLA) (from Grime *et al*., 2007), functional group and designated trait category

Species	SLA	Functional group	Category
*Deschampsia cespitosa* (Dc)	18.5	Grass	1
*Festuca rubra* (Fr)	17.7	Grass	1
*Poa pratensis* (Poap)	21.5	Grass	1
*Trisetum flavescens* (Tf)	20.1	Grass	1
*Campanula rotundifolia* (Cr)	22.2	Herb	1
*Filipendula ulmaria* (Fu)	18.6	Herb	1
*Leucanthemum vulgare* (Lv)	22.1	Herb	1
*Plantago lanceolata* (Pl)	22.4	Herb	1
*Anthoxanthum odoratum* (Ao)	27.3	Grass	2
*Cynosurus cristatus* (Cc)	26.4	Grass	2
*Dactylis glomerata* (Dg)	27.7	Grass	2
*Lolium perenne* (Lp)	26.4	Grass	2
*Centaurea nigra* (Cn)	26.3	Herb	2
*Geranium sylvaticum* (Gs)	NA^a^	Herb	2
*Hypochaeris radicata* (Hr)	23.3	Herb	2
*Ranunculus acris* (Ra)	23.8	Herb	2
*Agrostis capillaris* (Ac)	30.8	Grass	3
*Holcus lanatus* (Hl)	33.5	Grass	3
*Phleum pratense pratense* (Phlp)	30.6	Grass	3
*Poa trivialis* (Pt)	31.3	Grass	3
*Cerastium fontanum* (Cf)	29.2	Herb	3
*Leontodon hispidus* (Lh)	28.8	Herb	3
*Prunella vulgaris* (Pv)	30.3	Herb	3
*Rumex acetosa* (Ruma)	28.1	Herb	3

Category number indicates a gradient from conservative (category 1), to intermediate (category 2), and exploitative (category 3).

a SLA data not available for *G. sylvaticum* in Grime *et al*. (2007); unpublished data were used for including this species in category 2.

Each plant community consisted of both grasses and herbs from the same trait category, in equal abundances, to correct for functional group effects. We constructed plant communities consisting of species from one trait category only (two and four species mixtures), two trait categories in all possible combinations (two and four species mixtures), and three trait categories (six and 12 species mixtures; Table 2). Thus, our communities represented a range of CWM traits, as well as a gradient of species diversity, trait functional diversity and trait functional divergence. Species were randomly assigned to treatments. Each species occurred in nine plant communities and each of four replicate plant communities was unique, allowing us to investigate functional trait controls on N retention while controlling for individual species and functional group (grass vs herb) influences. This experimental design, including 14 treatments, each with four unique replicates, resulted in 56 different plant communities (Table 2).

**Table 2:** The 12 experimental treatments, representing a range of species richness treatments, number of categories (a proxy for functional diversity) and category average (a proxy for the ‘exploitativeness’ of the plant community, which averages the categories present, with 1 indicating dominance of conservative plant traits, and 3 indicating dominance of exploitative plant traits)

Treatment	No. of species	No. of categories	Categories	Average category
A	2	1	1	1
B			2	2
C			3	3
D		2	1 + 2	1.5
E			2 + 3	2.5
F			1 + 3	2
G	4	1	1	1
H			2	2
I			3	3
J		2	1 + 2	1.5
K			2 + 3	2.5
L			1 + 3	2
M	6	3	1 + 2 + 3	2
N	12	3	1 + 2 + 3	2

Each experimental treatment (A to N) had four unique replicates, drawn from the species pool in Table 1.

Seedlings of all species were germinated and grown for 13 wk (using the same soil that was used for the main experiment), after which they were transplanted into the experimental pots. Each plant community consisted of 12 individuals that were randomly assigned to a planting grid and planted at field densities. Pots were arranged in a randomized block design and kept at constant soil moisture (60% water‐holding capacity, which equalled 40% moisture content) in a controlled growth chamber (16 : 8 h, light : dark at 16°C) for 4.5 months.

After 4.5 months, 45 ml of ^15^NH_4_^15^NO_3_ solution (98.5 atom% enriched, 85 mg ^15^N, equivalent to 30 kg ha^−1^, which is the high end of yearly atmospheric N deposition in upland areas in the north of England (Defra, 2011), was injected in the top 5 cm at nine evenly spaced locations (5 ml each) of each pot, as in De Vries *et al*. (2012a). Forty‐eight hours after ^15^N addition, pots were leached by slowly adding 850 ml of demineralized water (equivalent to a 30 mm rainfall event), following the approach of De Vries *et al*. (2012a). We chose to harvest 48 h after N addition because a previous experiment showed that ecosystem N uptake at this time point is representative of longer‐term N retention: although initial N retention was predominantly in roots and microbes, this was later transferred to above‐ground plant parts, and as a result the total amount of N retained in plants, microbes and soil was the same after 48 h and after 2 months (De Vries *et al*., 2012a). Leachate volumes were recorded and leachates were kept in the fridge for a maximum of 1 wk until further analysis. Above‐ground vegetation was clipped and sorted to species, dried at 60°C for 48 h, weighed and ground. Soil was gently shaken off roots, passed through a 4 mm sieve and homogenized. Fresh soil was kept in the fridge until further analyses, and a subsample was air‐dried and ground. Roots were washed, dried at 60°C for 48 h, weighed and ground.

### C and N analyses

Leachates were analysed for inorganic N (NO_3_^−^ and NH_4_^+^), dissolved organic N (DON), and dissolved organic C (DOC), as described in De Vries *et al*. (2012a). Above‐ground vegetation (all species separately) and a representative subsample of roots were analysed for C and N using an Elementar Vario EL elemental analyzer (Hanau, Germany), and soil microbial biomass C and N were determined by fumigation extraction, as described by Brookes *et al*. (1985).

Leachate (after freeze‐drying), shoot (separated by species), root, soil and microbial biomass ^15^N (determined by diffusing microbial‐derived N onto an acid trap) were analysed using a Carlo Erba NA2000 analyser (CE Instruments, Wigan, UK) and a SerCon 20–20 isotope ratio mass spectrometer (SerCon Ltd, Crewe, UK) at Rothamsted Research, North Wyke. A dried and ground grass herbage sample labelled with ^15^N (2.79 atom% ^15^N) or natural abundance wheat flour (0.368 atom% ^15^N), both calibrated against IAEA‐N‐1 by Iso‐ Analytical (Crewe, UK), were used as the references for enriched or natural abundance samples, respectively. ^15^N excess atom% values, ^15^N concentrations in samples, total amounts of ^15^N in pools and total ecosystem ^15^N retention were calculated using the following calculations (De Vries *et al*., 2012a):

  $$\left(1\right)\;\;\text{atom}\%\;\text{excess}\;{}^{15}N=\text{atom}\%{}^{15}\text{N enriched}-\text{atom}\%{}^{15}\text{N natural abundance}$$
  $$\left(2\right)\;\;{}^{15}\text{N sample}\;\left(\text{mg}\;g^{-1}\right)=\text{atom}\%\;\text{excess}{}^{15}N\times \text{N sample}\;\left(\text{mg}\;g^{-1}\right)/100$$
  $$\begin{array}{cc} \;\left(3\right)\;\;{}^{15}\text{N pool}\;\left(\text{kg}\;{\text{ha}}^{-1}\right)= & \;{}^{15}\text{N sample}\;\left(\text{mg}\;g^{-1}\right)\times \text{pool}\;\left(g\;{\text{pot}}^{-1}\right)\\ & \times 352,698\;(\text{pots}\;{\text{ha}}^{-1})/1\\ & \times 10^{6}\;\left(\text{conversion from mg to kg}\right) \end{array}$$
  $$\left(4\right)\;\;{}^{15}\text{N retention}\;\left({\text{kgha}}^{-1}\right)={}^{15}\text{N shoot}+{}^{15}\text{N root}+{}^{15}\text{N soil}+{}^{15}\text{N microbes}\;(\text{all}\;\;\text{in}\;\;\text{kg}\;\;{\text{ha}}^{-1})$$

### Trait analyses

An additional set of monocultures of each plant species was grown under the same conditions (duration, planting density, soil type) in the experimental blocks of the main experiment for the analysis of leaf traits. Leaf trait analyses were not done on the main experiment because this would compromise the accuracy of ^15^N analysis, for which all above‐ground plant tissue is needed. One healthy leaf was cut under water from five individuals per species and rehydrated overnight below 6°C (Garnier *et al*., 2001). Leaf N content and C : N ratio, SLA and LDMC were measured using standard protocols (Cornelissen *et al*., 2003; Perez‐Harguindeguy *et al*., 2013). In addition, intact root systems of five individuals were washed and kept in 10% ethanol until analysis for specific root length (SRL), root diameter and root tissue density (RTD), using WinRhizo^®^ root analysis software (Regent Instruments Inc., Sainte‐Foy‐Sillery‐Cap‐Rouge, QC, Canada) and an Epson flatbed scanner. After analysis, roots were blotted dry, weighed, dried at 60°C for 48 h, and reweighed for root dry matter content (RDMC). Dry root samples were ground and analysed for C and N content using an Elementar Vario EL elemental analyzer.

Community‐weighted means for measured leaf functional traits were calculated using trait values per species and species relative abundance in treatments, assessed as DW (Garnier *et al*., 2004). In addition, trait functional diversity (Fd), trait functional divergence (FDiv), Rao's quadratic entropy (Rao), functional richness (FRich), and functional evenness (FEve) (Mouchet *et al*., 2010), were calculated using fdiversity software, as described by Casanoves *et al*. (2011). Although above‐ground plant community composition does not necessarily represent below‐ground species abundances, we also calculated CWM root traits based on root trait values per species.

### Statistical analyses

All data were checked for assumptions of normality and log‐transformed where necessary. We used linear mixed effect models (function lme in the R package nlme) to test species and functional group effects on species‐level trait measurements (with species as a nested factor to account for multiple measurements on one species). Species‐level root and leaf traits were examined by principal component analysis (PCA) using the R package vegan, and correlations between traits were analysed using Spearman's rank correlations. Treatment and plant community effects (number of categories, number of species and average category) on plant community attributes and N pools and retention were analysed using linear models (function lm in R). All analyses were done in R 3.2.0 (R Core Team, 2012).

**Figure 1:**
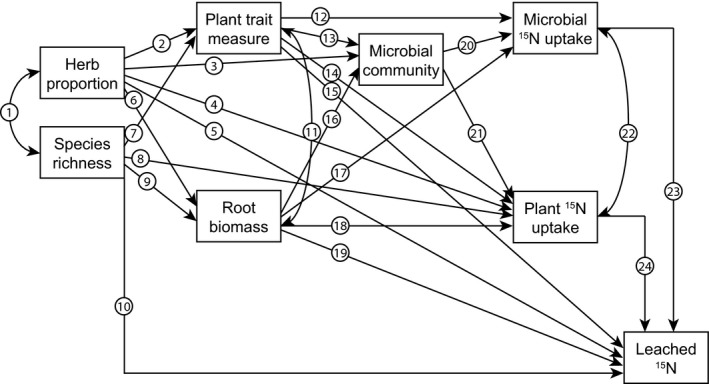
*A priori* model for ^15^N uptake by plants and microbes and ^15^N leaching. Arrow numbers are referred to below in bold. Herb proportion and species richness are allowed to covary (arrow **1**). Herb proportion can affect community‐weighted mean plant traits(**2**; see Fig. 3) and microbial community composition independently of traits through species‐specific effects (**3**; Harrison & Bardgett, 2010). Herb proportion can also directly affect plant ^15^N uptake (**4**; see Fig. 1) and nitrogen (N) leaching, by differing from grasses in above‐ground growth and evapotranspiration, and thus water uptake (**5**; Craine *et al*., 2002). Herbs can differ from grasses in their root biomass (**6**; Craine *et al*., 2002; Fujita *et al*., 2010). Species richness can affect plant trait measures through increased competition for light and resources (**7**; Roscher *et al*., 2012), and can increase plant N uptake through greater above‐ground biomass and growth (**8**; Tilman *et al*., 1996). Species richness can also increase root biomass through below‐ground overyielding (**9**; Ravenek *et al*., 2014), and can directly affect N leaching by greater above‐ground biomass and evapotranspiration, resulting in increased water uptake (**10**; Scherer‐Lorenzen *et al*., 2003). Plant trait measures can affect the microbial community through their effect on the quantity, quality and diversity of litter and C that is returned to the soil (Bardgett *et al*., 2014; Legay *et al*., 2014), and through direct associations with arbuscular mycorrhizal fungi, which might also serve as a conduit for plant‐derived C (**13**; Bardgett *et al*., 2014). Plant trait measures can affect plant and microbial N uptake and N leaching directly through below‐ground uptake of resources (**12**,** 14**,** 15**; De Vries *et al*., 2012a; Grassein *et al*., 2015). Plant traits and root biomass are allowed to covary because plant traits are strongly affected by plant size (**11**; Berendse & Moller, 2009; Craine *et al*., 2003). Root biomass can affect the microbial community by providing resources (rhizodeposits) (**16**; Orwin *et al*., 2010) and can affect microbial N uptake by competing for N (**17**). Root biomass can affect plant ^15^N uptake and N leaching through water and N uptake (**18**,** 19**; De Vries *et al*., 2012a). The microbial community affects N uptake through its composition and affinity for N (**20**; De Vries *et al*., 2012a; Myrold & Posavatz, 2007; Hodge & Fitter, 2010), and affects plant N uptake through competition for N, and through mycorrhizal N uptake (**21**; Mäder *et al*., 2000; Harrison *et al*., 2008; Hodge & Fitter, 2010). Microbes are stronger short‐term competitors for available N, and therefore microbial ^15^N uptake will affect plant ^15^N uptake (**22**; Harrison *et al*., 2008). Plant and microbial ^15^N uptake can both directly affect N leaching through decreasing the amount of soil available N that can be leached (**23**,** 24**; De Vries *et al*., 2012a).

**Figure 2:**
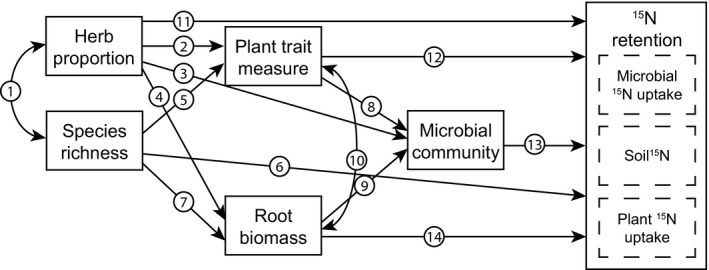
*A priori* model for ^15^N retention. Relationships between plant and microbial community properties are equal to those specified in Fig. 1 (arrows numbered 1–10); all plant and microbial community properties are hypothesized to influence ecosystem ^15^N retention through their effects on individual ^15^N pools, as specified in Fig. 1 (arrows numbered 11–14).

We performed structural equation modelling to test direct and indirect controls of plant community attributes, CWM traits, and trait diversity and divergence on ecosystem N pools and retention. This is a robust statistical method to test how experimental data fit a hypothesized causal structure that is well suited for investigating interactions between multiple traits and ecosystem functioning based on prior knowledge (Grace, 2006; Garcia‐Palacios *et al*., 2013). *A priori* models were constructed based on our hypotheses and theoretical knowledge of plant–microbe controls on N uptake and retention (Figs 1, 2). We selected plant community properties to be included based on their significance for explaining ^15^N pools in regression analyses, as detailed earlier. We first fitted models including only leaf traits, after which we fitted models including both leaf and root traits. Data were rescaled to correct for large differences in variances and we fitted our *a priori* models to the rescaled data using the lavaan package in R. We used model modification indices and stepwise removal of nonsignificant relationships, and tested the effect of these removals on Akaike information criterion (AIC) and model fit using a likelihood ratio test. We used a minimum set of parameters to assess model fit, including *χ* ^2^, root mean square error of approximation (RMSEA), and comparative fit index (CFI). Adequate model fits are indicated by a nonsignificant *χ* ^2^ test (*P *<* *0.05), high probability of a low RMSEA value (*P *>* *0.05) (Pugesek *et al*., 2003; Grace, 2006) and high CFI (> 0.95) (Byrne, 1994).

## Results

### Species‐level measurements

#### Leaf and root traits

Average leaf and root trait values are reported in Supporting Information Table S1. Leaf traits were better separated by PCA, and axes explained more variation in leaf than in root traits (Fig. S1). Species with high leaf N had low LDMC (Fig. S1a), while for root traits, species with high RTD and RDMC had low SRL and root N content (root N), respectively (Fig. S1b). Herbs clearly separated from grasses for leaf traits as well as root traits, with herbs generally having higher leaf N and lower LDMC, and lower SRL and RDMC than grasses (Fig. S2). Differences between herbs and grasses in individual traits were statistically significant for LDMC, leaf N, SRL and root N (Table 3).

**Table 3:** Mean trait values ± SE for grasses and herbs and *P*‐values for their difference (grasses, *n *=* *59; herbs, *n *=* *58)

	Grasses	Herbs	*P*‐value
LDMC (g g^−1^)	0.28 ± 0.01	0.17 ± 0.01	< 0.001
SLA (mm^2^ mg^−1^)	30.6 ± 1.3	31.3 ± 1.1	0.745
Leaf N (mg g^−1^)	13.2 ± 0.4	19.3 ± 0.5	0.030
RDMC (g g^−1^)	0.29 ± 0.03	0.21 ± 0.02	0.087
SRL (cm g^−1^)	29638 ± 772	17609 ± 1407	0.002
Root N (mg g^−1^)	7.3 ± 0.1	9.7 ± 0.4	0.011
RTD (g cm^−3^)	0.17 ± 0.01	0.20 ± 0.02	0.422

LDMC, leaf dry matter content; SLA, specific leaf area; leaf N, leaf N content; RDMC, root dry matter content; SRL, specific root length; root N, root N content; RTD, root tissue density.

Across all species, leaf N was correlated positively with SLA and negatively with LDMC, while RTD was correlated positively with RDMC and negatively with root N (Table 4). The leaf trait SLA was positively correlated with the root trait RTD, while leaf N was negatively correlated with SRL. LDMC and RDMC were strongly positively correlated (Table 4).

**Table 4:** Spearman's rank correlation matrix of plant traits measured for all 24 species occurring in the experimental treatments (*n *=* *117). Values indicate *R* values; values in bold are *P *<* *0.05

	LDMC	SLA	Leaf N	RDMC	SRL	Root N	RTD
LDMC		−0.18	−**0.57**	**0.47**	0.27	−0.36	0.14
SLA			**0.46**	0.34	−0.02	−0.02	**0.53**
Leaf N				−0.13	−**0.44**	0.20	0.26
RDMC					−0.03	−0.40	**0.68**
SRL						0.11	−0.39
Root N							−**0.45**
RTD							

LDMC, leaf dry matter content; SLA, specific leaf area; leaf N, leaf N content; RDMC, root dry matter content; SRL, specific root length; root N, root N content; RTD, root tissue density.

#### Species‐specific ^15^N uptake

**Figure 3:**
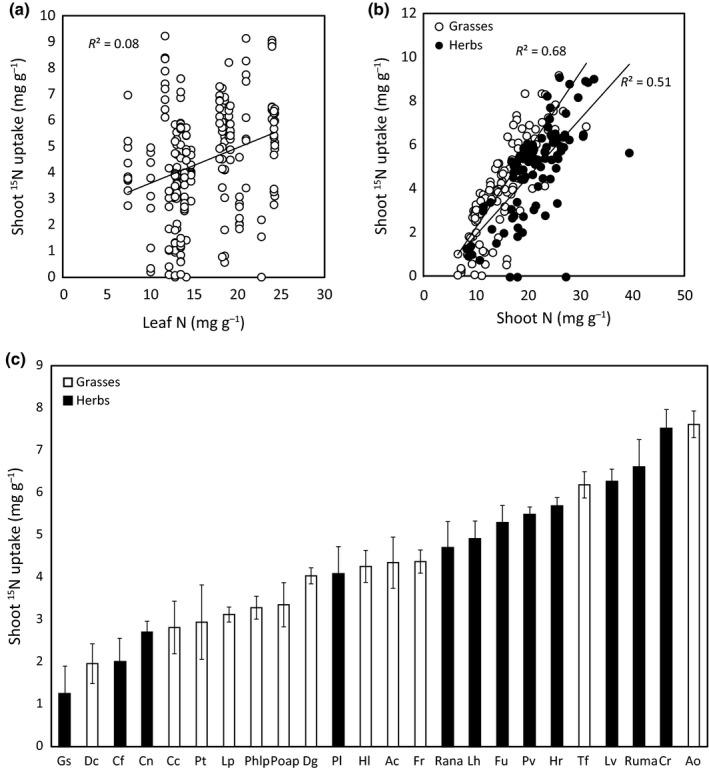
Species‐specific ^15^N uptake as explained by the species‐level trait leaf nitrogen (N) content (a), actual shoot N content (b) and species identity (c), for grasses (white) and herbs (black). Symbols represent individual observations (a,b); bars represent means ± 1 SE (*n* varies between 3 and 12). For abbreviations of species names in (c) see Table 1.

Shoot ^15^N uptake varied strongly across species (Fig. 3c). We did not find an effect of category rank, category number or species richness of the assembled plant community on ^15^N uptake by individual species, nor could individual species uptake of ^15^N be explained by species‐level root traits. There was a weak trend of increasing uptake of ^15^N by individual species with greater species‐level leaf N (*P *<* *0.0001, *R* ^2^ = 0.08, respectively; Fig. 3a). However, the best predictor for species‐specific shoot ^15^N uptake was shoot N content (*F*_1,211_ = 301.1, *P *<* *0.001; Fig. 3b), and this relationship differed between grasses and herbs (*F*_1,211_ = 7.06, *P* = 0.009; Fig. 3b). Herbs took up more ^15^N than grasses overall (Fig. 3c) and had greater shoot N content, but they took up less ^15^N per unit shoot N than grasses (Fig. 3b).

### Treatment effects on plant community attributes

The only trait we found to increase with category rank was CWM SLA (*P *<* *0.0001, *R* ^2^ = 0.37; Fig. S3a). We did not find any change of CWM root traits with category rank (Table 5). Still, as intended, our constructed plant communities represented a range of CWM functional trait values, for both leaf and root traits (Fig. S4; Table S2). Both CWM leaf N and CWM root N values were positively correlated with shoot N content and root N content of total above‐ground and below‐ground vegetation (Fig. S5), but were overestimating actual N content of these pools. This was more apparent for CWM root N, which also had a considerably lower predictive power (Fig. S5). As a result of differences between grasses and herbs for LDMC, leaf N, SRL and root N (Table 3; Fig. S2), CWM values of these traits were strongly affected by the proportion of herb biomass of total above‐ground biomass (Fig. S6). The proportion of herb biomass itself was not affected by our treatments (Table 5).

**Table 5:** Statistics for linear models of treatment effects on plant community properties

Predictor	Response variable	*R* ^2^	*P*‐value
Species richness	Above‐ground biomass	0.006	0.565
	Root biomass	**0.079**	**0.036**
	Herb proportion	0.008	0.507
	Functional diversity	**0.334**	**< 0.001**
	Functional divergence	0.014	0.383
	Functional richness	**0.295**	**<0.001**
	Rao's quadratic entropy	**0.117**	**0.010**
	Evenness	0.027	0.228
	Shannon's diversity	**0.719**	**< 0.001**
Nr of categories	Above‐ground biomass	< 0.001	0.998
	Root biomass	0.026	0.232
	Herb proportion	0.010	0.446
	Functional diversity	**0.138**	**0.0049**
	Functional divergence	< 0.001	0.975
	Functional richness	0.048	0.105
	Functional evenness	0.006	0.648
	Rao's quadratic entropy	0.030	0.201
	Evenness	0.022	0.279
	Shannon's diversity	**0.266**	**< 0.001**
Category average	Above‐ground biomass	0.001	0.861
	Root biomass	0.001	0.771
	Herb proportion	0.010	0.463
	CWM SLA	**0.363**	**< 0.001**
	CWM LDMC	0.028	0.211
	CWM leaf N	0.004	0.662
	CWM SRL	0.004	0.644
	CWM RDMC	0.067	0.055
	CWM root N	0.021	0.286
	CWM RTD	0.042	0.131

For minimum, maximum and average values for these properties see Supporting Information Table S2.

Values in bold are *P* < 0.05.

CWM, community‐weighted mean; LDMC, leaf dry matter content; SLA, specific leaf area; leaf N, leaf N content; RDMC, root dry matter content; SRL, specific root length; root N, root N content; RTD, root tissue density.

We found that functional diversity increased with both number of categories (*P* = 0.0049, *R* ^2^ = 0.14; Fig. S3b; Table 5) and realized species richness (*P *<* *0.0001, *R* ^2^ = 0.33; Fig. S3d; Table 5); root biomass increased weakly with species richness (*P* = 0.036, *R* ^2^ = 0.08; Fig. S3c; Table 5); and Rao's quadratic entropy also increased weakly with species richness (*P* = 0.010, *R* ^2^ = 0.12; Fig. S3e; Table 5). By contrast, functional richness decreased with species richness (*P *<* *0.0001, *R* ^2^ = 0.30; Fig. S3f; Table 5), and above‐ground biomass was not affected by our treatments (Table 5).

### Effects of plant traits and community attributes on ^15^N pools and retention

**Figure 4:**
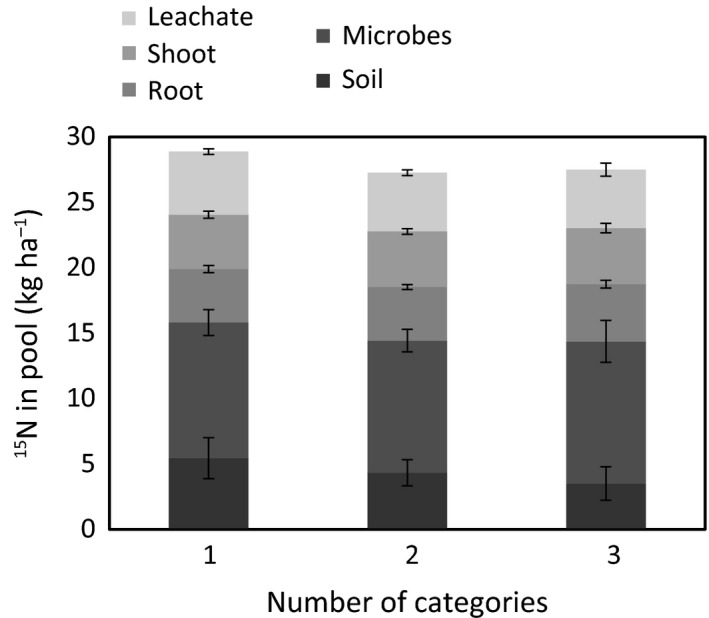
Uptake of ^15^N in the various ecosystem pools. The size of ^15^N pools was not affected by the number of categories. Bars represent treatment means ± 1 SE (*n* = 24 for one and two categories, *n* = 8 for three categories).

**Figure 5:**
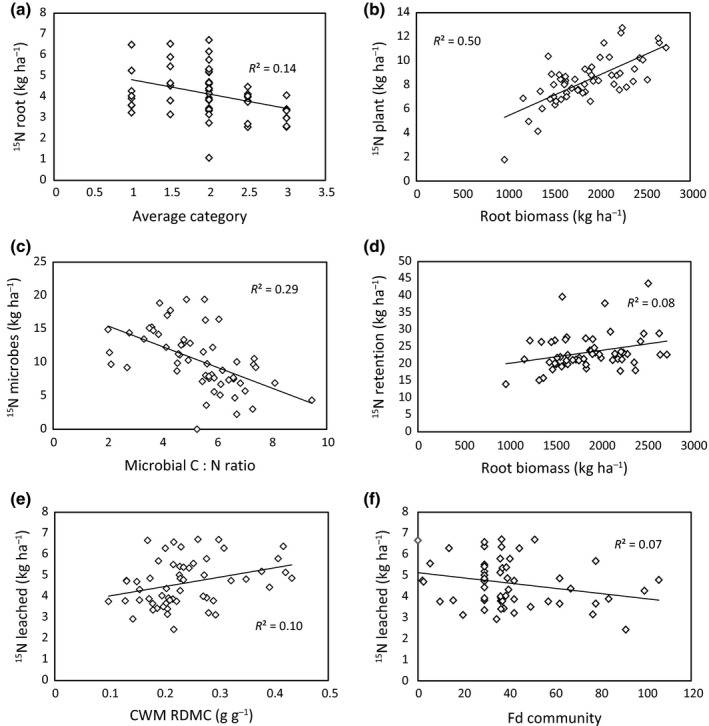
Relationships of plant and microbial community attributes with ^15^N pools. (a) Root ^15^N uptake decreased with higher category rank; (b) plant ^15^N uptake increased with greater root biomass; (c) microbial ^15^N uptake decreased with increasing microbial biomass C : N ratio; (d) ^15^N retention in plant and soil pools increased with root biomass; (e, f) ^15^N leached increased with community‐weighted mean (CWM) root dry matter content (RDMC) (e) and decreased with functional diversity (Fd) (d). Symbols represent individual observations. See text for statistics.

The greatest amount of added ^15^N was taken up by soil microbes, followed by plant tissue (root and shoot) and soil (Fig. 4). These pools were not affected by the plant community treatments (category rank, number of categories and species richness), although root uptake of ^15^N decreased with greater category rank (i.e. communities constructed to be dominated by exploitative traits, *P* = 0.004, *R* ^2^ = 0.12; Fig. 5a). Although treatment effects on ^15^N pools and retention were limited, several plant and microbial community attributes were related to ^15^N pools and retention. Total plant ^15^N uptake increased with root biomass (*P *<* *0.001, *R* ^2^ = 0.50; Fig. 5b). Microbial uptake of ^15^N decreased with microbial C : N ratio (*P *<* *0.001, *R* ^2^ = 0.29; Fig. 5c), and the retention of ^15^N in the plant–soil system increased, albeit weakly, with root biomass (*P* = 0.07, *R* ^2^ = 0.08; Fig. 5d). Finally, the amount of ^15^N leached from the system increased with CWM RDMC and decreased with functional diversity (*P* = 0.016, *R* ^2^ = 0.10; Fig. 5e; and *P* = 0.05, *R* ^2^ = 0.07; Fig. 5f). Herb biomass increased both root and shoot uptake of ^15^N (*P* = 0.0010, *R* ^2^ = 0.18 and *P* = 0.0014, *R* ^2^ = 0.17, respectively; Fig. S7).

All inorganic N that was leached consisted of the added ^15^N (*P *<* *0.001, *R* ^2^ = 0.84; Fig. S8a), and the amount of ^15^N leached was strongly positively linked to the amounts of DON and DOC leached (*P *<* *0.001, *R* ^2^ = 0.25, and *P* = 0.006, *R* ^2^ = 0.13, respectively; Fig. S8b,c). The amounts of ^15^N leached, inorganic N leached and DOC leached all significantly increased with greater CWM LDMC and RDMC (Fig. S9); DON leached was not explained by any plant community properties. The only system ^15^N pool that significantly explained the total amount of ^15^N retained in the system was soil ^15^N (*P *<* *0.001, *R* ^2^ = 0.44), which itself was not explained by any plant community properties (Fig. S10).

**Figure 6:**
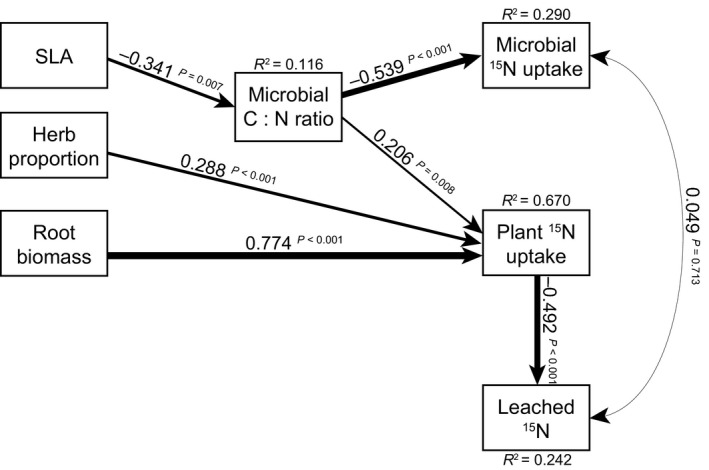
Our final, most parsimonious model for explaining ecosystem ^15^N pools and leaching, using community‐weighted mean (CWM) leaf traits. The weight of the arrows indicates the strength of the causal relationship, supplemented by a standardized path coefficient and *P*‐value. *R* ^2^ values denote the amount of variance explained by the model for the response variables. The model fitted the data well (*χ* ^2^ = 11.014, df = 11, *P* = 0.442; comparative fit index = 1.000; root mean square error of approximation < 0.05, *P* = 0.563, Akaike information criterion = 2516.5). See Supporting Information Tables S3 and S4 for details on model selection and partial *R* ^2^ values. SLA, specific leaf area.

Our structural equation models (SEMs) revealed that plant traits and plant community attributes both directly and indirectly controlled ^15^N uptake in the various ecosystem pools, and the amount of ^15^N retained in the plant–soil system after leaching. The SEM for explaining ^15^N leaching and plant and microbial ^15^N pools only using leaf traits fitted the data well (*χ* ^2^ = 11.014, df = 11, *P* = 0.442; CFI = 1.000; RMSEA < 0.05, *P* = 0.563), and showed that root biomass and the proportion of herbs directly controlled plant ^15^N uptake, while CWM SLA indirectly controlled both plant and microbial ^15^N uptake through its effect on microbial biomass C : N ratio (Fig. 6). Root biomass strongly increased plant uptake, which subsequently decreased ^15^N leached (the standardized indirect effect of root biomass on ^15^N leached = 0.774 × −0.492 = −0.381). The proportion of herbs in above‐ground biomass also decreased N leaching through its effect on plant ^15^N uptake, although this indirect effect was weaker than that of root biomass on ^15^N leaching (indirect effect of herb proportion on ^15^N leached = 0.288 × −0.492 = −0.142). Higher CWM SLA decreased the microbial C : N ratio, which in turn increased microbial ^15^N uptake (indirect effect of SLA on microbial ^15^N = −0.341 × −0.539 = 0.183), and decreased plant ^15^N uptake (indirect effect of SLA on plant ^15^N = −0.341 × 0.206 = −0.070).

**Figure 7:**
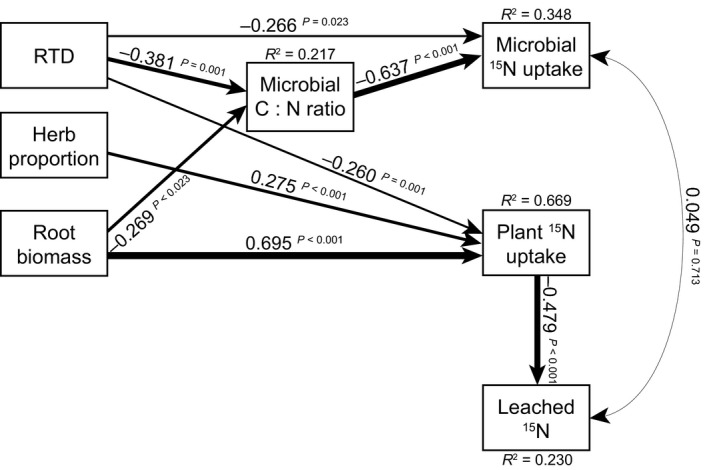
Our final, most parsimonious model for explaining ecosystem ^15^N pools and leaching, using both community‐weighted mean (CWM) leaf and root traits. The weight of the arrows indicates the strength of the causal relationship, supplemented by a path coefficient. *R* ^2^ values denote the amount of variance explained by the model for the response variables. The fit of this model was good (*χ* ^2^ = 7.225, df = 9, *P* = 0.614; comparative fit index = 1.000; root mean square error of approximation < 0.05, *P* = 0.708, Akaike information criterion = 2546.4). See Supporting Information Tables S5 and S6 for details on model selection and partial *R* ^2^ values. RTD, root tissue density.

When we used both leaf and root traits in our model explaining ^15^N leaching and plant and microbial ^15^N pools, RTD was retained as a significant predictor, while SLA dropped out (Table S5). However, although RTD was a better predictor for ^15^N pools than SLA, still the model including RTD had a higher AIC than our model including only SLA (2546.4 for the model including RTD vs 2516.5 for the model including SLA; Figs 6 and 7). This model included many similar relationships to that including only SLA; however, one major difference was that plant ^15^N uptake decreased with a higher CWM RTD (Fig. 7). In addition, apart from a higher RTD decreasing microbial C : N ratio and indirectly increasing microbial ^15^N uptake (indirect effect of RTD on microbial ^15^N = −0.365 ×  −0.623 = 0.227), a higher RTD also directly decreased microbial ^15^N uptake. Still, root biomass was the best predictor for plant ^15^N uptake, and indirectly for ^15^N leaching (indirect effect of root biomass on ^15^N leached = 0.680 × −0.480 = −0.326; Fig. 7).

**Figure 8:**
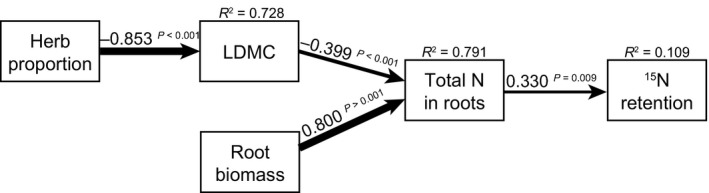
Our final, most parsimonious model for explaining ecosystem ^15^N retention, using both community weighted mean (CWM) leaf and root traits. The weight of the arrows indicates the strength of the causal relationship, supplemented by a path coefficient. *R* ^2^ values denote the amount of variance explained by the model for the response variables. The fit of this model was good (*χ* ^2^ = 4.309, df = 5, *P* = 0.506; comparative fit index = 1.000; root mean square error of approximation < 0.05, *P* = 0.582). See Supporting Information Tables S7 and S8 for details on model selection and partial *R* ^2^ values. LDMC, leaf dry matter content.

In addition to testing our *a priori* SEMs explaining plant and microbial ^15^N uptake and leaching of ^15^N, we used SEM to test which plant community properties explained ^15^N retention in the plant–soil system, which is the sum of ^15^N retained in plants, microbes and soil (Fig. 2). Our final model for ^15^N retention (the sum of plant, microbial and soil ^15^N retained in the system after leaching) only included a few predictors (Fig. 8) – this model did not change when including both leaf and root traits. The fit of this model was good (*χ* ^2^ = 4.309, df = 5, *P* = 0.506; CFI = 1.000; RMSEA < 0.05, *P* = 0.582), with ^15^N retention being strongly linked to the total root N pool, which in turn was affected by LDMC and root biomass; LDMC content was reduced by the proportion of herbs in above‐ground biomass. This lower LDMC increased the total root N pool and thus indirectly ^15^N retention (indirect effect of herb proportion on ^15^N retention = −0.853 × −0.399 × 0.330 = 0.112, and of LDMC on ^15^N retention = −0.399 × 0.330 = −0.132). Greater root biomass indirectly increased ^15^N retention through its positive effect on the total root N pool (indirect effect of root biomass on ^15^N retention = −0.800 × 0.330 × 0.33 = 0.264).

## Discussion

Our experimental treatments created a gradient of CWM leaf traits, functional diversity and divergence representative of those found in the field. For example, in a previous study covering a range of grassland types across England, CWM SLA ranged from 17.6 to 35.1 mm^2^ mg^−1^, CWM LDMC from 0.15 to 0.35 g g^−1^, and CWM leaf N from 17.8 to 35.1 mg g^−1^ (De Vries *et al*., 2012b). In comparison, in our treatments, CWM SLA ranged from 19.8 to 41.8 mm^2^ mg^−1^, CWM LDMC ranged from 0.14 to 0.33 g g^−1^, and CWM leaf N ranged from 7.57 to 20.89 mg g^−1^. Given this, the gradients in CWM leaf traits produced in our study allowed us to test our contrasting hypotheses on plant community controls on ecosystem N retention. We hypothesized that either the dominance of conservative leaf traits controls plant and microbial N uptake and hence N leaching loss and ecosystem N retention, or that trait functional diversity or divergence enhanced N retention through greater plant N uptake. We found that root biomass, the proportion of herbs in communities, dominant leaf traits and, to a lesser extent, dominant root traits controlled ^15^N uptake by plants and microbes, and ^15^N leached. Thus, our results support the mass‐ratio hypothesis, rather than the diversity hypothesis.

Although root biomass only increased marginally with higher species richness, greater root biomass significantly increased plant ^15^N uptake and indirectly increased microbial ^15^N uptake, reducing the amount of ^15^N leached and increasing ^15^N retention in the plant–soil system. The proportion of herbs in our plant communities increased plant ^15^N uptake, while a higher CWM SLA indirectly increased microbial ^15^N uptake, and a higher CWM RTD decreased plant ^15^N uptake. These results confirm the central role of roots in ecosystem N retention (De Vries *et al*., 2012a), and corroborate findings that plants with exploitative growth strategies have the highest rates of N uptake (Grassein *et al*., 2015). However, they contradict the notion (De Vries & Bardgett, 2012), and field observations (Laughlin, 2011; De Vries *et al*., 2012a; Grigulis *et al*., 2013), that plant communities dominated by slow‐growing, resource‐conservative species and their associated microbial communities have the greatest N retention.

Our treatments created a wide gradient in CWM SLA, which was also the trait included in our SEM for explaining plant and microbial ^15^N uptake and ^15^N leaching. CWM SLA indirectly increased microbial ^15^N uptake through modifying the microbial C : N ratio. Greater CWM SLA decreased the microbial C : N ratio, apparently alleviating microbial N limitation and potentially indicating a shift towards more bacterial‐dominated microbial communities, which are characterized by a lower C : N ratio than fungal‐dominated communities (Van Veen & Paul, 1979; Bloem *et al*., 1997). This link between exploitative plant traits and C‐limited, bacterial‐dominated microbial communities supports similar findings from field observations (Orwin *et al*., 2010; De Vries *et al*., 2012b; Grigulis *et al*., 2013). These linkages between plant traits and microbial communities are often attributed to the quality and quantity of plant litter inputs (Bardgett & Wardle, 2010), but the duration of our experiment was too short to allow for significant litter inputs. Therefore, it is more likely that root processes influenced microbial communities. We found that CWM RTD affected microbial C : N ratio and microbial ^15^N uptake in the same direction as CWM SLA. This follows the positive correlation we found between these two traits, but is contrary to our expectation, as higher RTD indicates a greater investment in tissue longevity and efficient C use, and would thus be placed towards the conservative end of the root economics spectrum. By contrast, the decreased plant ^15^N uptake with higher CWM RTD suggests that this trait is associated with conservative growth strategies.

In contrast to our expectation, lower microbial C : N ratio was associated with greater microbial ^15^N uptake, indicating that despite an alleviation of N limitation, these microbes had the greatest affinity for N. This might indicate a shift to greater relative abundance of bacteria, which have been suggested to be able to use larger amounts of readily available N than fungi (Myrold & Posavatz, 2007), despite many studies reporting greater ^15^N immobilization in fungal‐dominated microbial communities (De Vries *et al*., 2011, 2012a). Our results therefore indicate that soil microbial communities that are not N‐limited have the greatest affinity for available N and can increase N retention in the plant–soil system, especially given that the ^15^N immobilized in microbes exceeded that in plants (Fig. 5). Our SEM including CWM SLA shows that a lower microbial C : N ratio decreased plant ^15^N uptake, indicating an intensified competition for N between plants and microbes, as also found by Moreau *et al*. (2015). Although we found no direct competition between plant and microbial ^15^N pools, this is supported by our finding that CWM RTD increased microbial ^15^N, but decreased plant ^15^N uptake.

The proportion of herbs in our plant communities was an important determinant of plant ^15^N uptake, and thus indirectly of ^15^N retention. Total plant ^15^N uptake was higher with an increased proportion of herbs, and on an individual plant level, herbs had higher shoot ^15^N uptake than grasses. This is in contrast to previous findings of greater N allocation into root and shoot biomass in grasses compared with herbs (Robson *et al*., 2010), and of reduced N leaching with higher grass abundance, which has been attributed to their thin and dense root systems (Phoenix *et al*., 2008; De Vries *et al*., 2015). Indeed, we found that herbs had higher SRL than grasses, but this was not the trait that explained plant ^15^N uptake on a community or individual species level. Herbs also had lower LDMC and higher root and leaf N than grasses, of which leaf N might underlie their higher uptake of ^15^N, as this trait best explained individual species ^15^N uptake. In addition, higher LDMC reduced the root N pool in our plant communities, which in turn reduced ecosystem ^15^N retention. These findings are in line with the findings by Grassein *et al*. (2015), who found that the uptake and affinity for N of individual grasses increased with exploitative leaf traits. Importantly, although herbs differed from grasses in most traits and thus affected CWM values of these traits in our mixtures, they did not differ in SLA and RTD, which were the traits that best explained plant and microbial ^15^N uptake in our SEMs.

Greater species richness did not result in greater above‐ground biomass, but it did result in overyielding below ground (Ravenek *et al*., 2014), and this greater root biomass was associated with a reduced microbial C : N ratio, which in turn increased microbial ^15^N uptake. Greater root biomass also strongly increased plant ^15^N uptake (Figs 3b, 5, 6) and the total root N pool (Fig. 8), and hence ^15^N retention (Fig. 3d). Root N was increased with lower CWM values of the conservative trait LDMC, and this greater root N pool increased ^15^N retention (Fig. 8). These results corroborate previous studies that show the importance of root N uptake for ecosystem N retention (Zogg *et al*., 2000; De Vries *et al*., 2012a, 2015). Moreover, they point to the dominance of exploitative plant traits, namely low CWM LDMC and high root N content, enhancing ecosystem N retention. This is in line with results from Garcia‐Palacios *et al*. (2013), who found, in a pot experiment similar in scale and duration to ours, that CWM SLA reduced soil N availability. Our results suggest that greater root and shoot uptake in plant communities dominated by conservative species, as reported in field observations (De Vries *et al*., 2012a; Hoeft *et al*., 2014; but see Bingham & Biondini, 2011), might be a result of low nutrient availability, rather than of a greater affinity for N of slow‐growing, resource‐conservative plant species.

The total amount of ^15^N retained in our system consisted of the sum of ^15^N in plant, microbes, and soil. Although ^15^N uptake in plants and microbes, as well as the amount of ^15^N leached from the system, were well‐explained by root biomass, the proportion of herbs, and CWM leaf and root traits, we struggled to find adequate predictors of ecosystem ^15^N retention. We found that the total amount of ^15^N retained was best explained by the amount of ^15^N that was retained in soil (Fig. S10), which in itself was a highly variable pool that was not related to any plant community attributes. In addition, there was much unexplained variation in many of our measured ^15^N pools. Since our soil was sieved, homogenized, and packed to the same bulk density across all pots, we do not believe that this unexplained variation was caused by differences in soil texture, density, pH, or organic matter content, which all play a role in the adsorption of positively charged ions such as ammonium (Six *et al*., 1998; Denef *et al*., 2002; Gonod *et al*., 2006). However, this unexplained variation might point to the importance of particular groups of microbes and their activities, which can be linked to plant functional traits, for explaining variation in ^15^N pools (e.g. Cantarel *et al*., 2015; Moreau *et al*., 2015).

Despite the need for caution in calculating CWM root traits based on above‐ground species abundances, we found that several CWM root traits affected plant and microbial ^15^N uptake, but our SEM including CWM SLA was superior to that including CWM RTD. This might be a result of the discrepancy between above‐ground and below‐ground community composition rather than root traits actually being worse predictors for these pools, as several studies have found that root traits have a stronger control on ecosystem N dynamics and retention than above‐ground functional traits, which were the focus of our study (Grigulis *et al*., 2013; Bardgett *et al*., 2014; but see Grassein *et al*., 2015). The correlations we found between leaf and root traits support previous work (Craine *et al*., 2005; Tjoelker *et al*., 2005; Roumet *et al*., 2006; Freschet *et al*., 2010); however, they do not support the existence of a root economics spectrum, as above‐ground exploitative traits correlated strongly with below‐ground traits considered to be conservative.

Collectively, our results show that root biomass, herb abundance and the dominance of exploitative leaf traits, namely high SLA and leaf N and low LDMC, directly and indirectly increase short‐term ecosystem N retention. However, caution is needed when interpreting these results: plant communities dominated by fast‐growing, resource‐exploitative species and their associated microbial communities might rapidly take up available N, but high rates of nutrient cycling also mean that N is remineralized (Bengtson & Bengtsson, 2005) and potentially lost quickly from ecosystems. We did not measure this process, but it can be relevant at longer timescales. Nevertheless, our results show that N addition increases N uptake by exploitative plants and microbes, thereby possibly favouring their dominance in the longer term. In sum, we show that dominant plant traits, rather than trait functional diversity, contribute to the fate of added N in the plant–soil system.

## Author contributions

F.T.d.V and R.D.B planned and designed the research; F.T.d.V. performed the experiment and analysed the data; and F.T.d.V. and R.D.B. wrote the manuscript.

## Supporting information

Please note: Wiley Blackwell are not responsible for the content or functionality of any supporting information supplied by the authors. Any queries (other than missing material) should be directed to the *New Phytologist* Central Office.

**Fig. S1** PCA biplots for leaf traits and root traits for the 24 species used in the experiment.
**Fig. S2** PCA biplots for leaf traits and root traits for the 24 species used in the experiment.
**Fig. S3** Treatment (average trait category, number of trait categories, and species richness; see Tables 1 and 2) effects on plant community attributes and ^15^N pools.
**Fig. S4** Histograms showing frequency distributions for community‐weighted mean (CWM) leaf and root traits for the experimental communities.
**Fig. S5** The relationship between community‐weighted mean (CWM) leaf N and root N content calculated from individual abundances and species‐averaged traits and measured total community shoot and root N content.
**Fig. S6** The effect of the proportion of herb biomass of total community biomass on values for CWM traits, for leaf and root traits.
**Fig. S7** Relationships between above‐ground ^15^N uptake and herb biomass.
**Fig. S8** Relationship between ^15^N leached and the amounts of inorganic N, dissolved organic N (DON), and dissolved organic C (DOC) leached.
**Fig. S9** Amounts of ^15^N, DON, inorganic N, and DOC leached as explained by leaf dry matter content (LDMC) and root dry matter content (RDMC).
**Fig. S10** Relationships between individual ^15^N pools and the amount of ^15^N retained in the system.
**Table S1** Leaf and root trait values per species
**Table S2** Minimum, maximum and mean values for plant community attributes in our experiment
**Table S3** Model selection procedure and statistics for the structural equation model (SEM) explaining ^15^N pools and leaching, only including leaf traits
**Table S4** The effect on *R* ^2^ of the removal of individual parameters from regressions containing multiple predictors in the final SEM for ^15^N pools and leaching, only including leaf traits
**Table S5** Model selection procedure and statistics for the structural equation model (SEM) explaining ^15^N pools and leaching, including leaf traits as well as root traits
**Table S6** The effect on *R* ^2^ of the removal of individual parameters from regressions containing multiple predictors in the final SEM for ^15^N pools and leaching, including leaf and root traits
**Table S7** Model selection procedure and statistics for the structural equation model (SEM) explaining ^15^N retention, including leaf traits as well as root traits
**Table S8** The effect on *R* ^2^ of the removal of individual parameters from regressions containing multiple predictors in the final SEM for ^15^N retention
