# Supplementary material for: Plant community controls on short‐term ecosystem nitrogen retention
Source: New Phytol. 2016 Jan 8;210(3):861–74. doi: 10.1111/nph.13832 (PMC4981912; doi:10.1111/nph.13832)
Supplement: Supplementary file 1 — Fig. S1 PCA biplots for leaf traits and root traits for the 24 species used in the experiment. Fig. S2 PCA biplots for leaf traits and root traits for the 24 species used in the experiment. Fig. S3 Treatment (average trait category, number of trait categories, and species richness; see Tables 1 and 2) effects on plant community attributes and 15N pools. Fig. S4 Histograms showing frequency distributions for community‐weighted mean (CWM) leaf and root traits for the experimental communities. Fig. S5 The relationship between community‐weighted mean (CWM) leaf N and root N content calculated from individual abundances and species‐averaged traits and measured total community shoot and root N content. Fig. S6 The effect of the proportion of herb biomass of total community biomass on values for CWM traits, for leaf and root traits. Fig. S7 Relationships between above‐ground 15N uptake and herb biomass. Fig. S8 Relationship between 15N leached and the amounts of inorganic N, dissolved organic N (DON), and dissolved organic C (DOC) leached. Fig. S9 Amounts of 15N, DON, inorganic N, and DOC leached as explained by leaf dry matter content (LDMC) and root dry matter content (RDMC). Fig. S10 Relationships between individual 15N pools and the amount of 15N retained in the system. Table S1 Leaf and root trait values per species Table S2 Minimum, maximum and mean values for plant community attributes in our experiment Table S3 Model selection procedure and statistics for the structural equation model (SEM) explaining 15N pools and leaching, only including leaf traits Table S4 The effect on R 2 of the removal of individual parameters from regressions containing multiple predictors in the final SEM for 15N pools and leaching, only including leaf traits Table S5 Model selection procedure and statistics for the structural equation model (SEM) explaining 15N pools and leaching, including leaf traits as well as root traits Table S6 The effect on R 2 of the removal of individual paramete [file NPH-210-861-s001.pdf]

New Phytologist

New Phytologist **Supporting Information**

Article title: Plant community controls on short-term ecosystem nitrogen retention

Authors: Franciska T. de Vries and Richard D. Bardgett

Article acceptance date: 27 November 2015

The following Supporting Information is available for this article.

Table S1 Leaf and root trait values per species

Table S2 Minimum, maximum, and mean values for plant community attributes in our experiment

Table S3 Model selection procedure and statistics for the structural equation model (SEM) explaining ^{15}N pools and leaching, only including leaf traits

Table S4 The effect on *R*-squared of the removal of individual parameters from regressions containing multiple predictors in the final SEM for ^{15}N pools and leaching, only including leaf traits

Table S5 Model selection procedure and statistics for the structural equation model (SEM) explaining ^{15}N pools and leaching, including leaf traits as well as root traits

Table S6 The effect on *R*-squared of the removal of individual parameters from regressions containing multiple predictors in the final SEM for ^{15}N pools and leaching, including leaf and root traits

Table S7 Model selection procedure and statistics for the structural equation model (SEM) explaining ^{15}N retention, including leaf traits as well as root traits

Table S8 The effect on *R*-squared of the removal of individual parameters from regressions containing multiple predictors in the final SEM for ^{15}N retention

Fig. S1 PCA biplots for leaf traits (a) and root traits (b) for the 24 species used in the experiment.

Fig. S2 PCA biplots for leaf traits (a) and root traits (b) for the 24 species used in the experiment.

Fig. S3 Treatment (average trait category, number of trait categories, and species richness, see Tables 1 and 2) effects on plant community attributes and ^{15}N pools.

Fig. S4 Histograms showing frequency distributions for community weighted mean (CWM) leaf and root traits for the experimental communities.

Fig. S5 The relationship between community weighted mean (CWM) leaf N and root N content calculated from individual abundances and species averaged traits and measured total community shoot and root N content.

Fig. S6 The effect of the proportion of herb biomass of total community biomass on values for CWM traits, for leaf traits and root traits.

Fig. S7 Relationships between aboveground ^{15}N uptake and herb biomass.

Fig. S8 Relationship between ^{15}N leached and the amounts of inorganic N, dissolved organic N (DON), and dissolved organic C (DOC) leached.

Fig. S9 Amounts of ^{15}N, DON, inorganic N, and DOC leached as explained by leaf dry matter content (LDMC) and root dry matter content (RDMC).

Fig. S10 Relationships between individual ^{15}N pools and the amount of ^{15}N retained in the system.

Table S1 Leaf and root trait values per species (mean±SE, except for leaf N, which was measured on pooled leaves of all individuals; n=5 for all species except *Agrostis capillaris* (*n*=4) and *Filipendula ulmaria* (*n*=3)). These values were used for calculating CWM traits values for the main experiment.

Species	LDMC (g g^{-1})	SLA (mm^2 mg^{-1})	LeafN (mg g^{-1})	RDMC (g g^{-1})	SRL (cm g^{-1})	RootN (mg g^{-1})	RTD (g cm^{-3})
Agrostis capillaris	0.28±0.03	39.7±2.9	18.60	0.19±0.01	28159±2563	6.23±0.30	0.19±0.02
Anthoxanthum odoratum	0.30±0.01	28.3±1.2	11.70	0.25±0.02	35617±1336	6.69±0.22	0.13±0.00
Cynosurus cristatus	0.27±0.01	24.3±1.5	10.10	0.17±0.01	25136±1390	6.80±0.12	0.13±0.00
Cerastium fontanum	0.16±0.01	37.1±2.2	12.90	0.29±0.06	29047±1749	14.87±1.44	0.13±0.01
Centaurea nigra	0.14±0.01	33.9±1.3	20.30	0.15±0.01	8665±728	7.50±0.26	0.19±0.01
Campanula rotundifolia	0.20±0.00	33.7±1.2	24.00	0.12±0.01	41461±1342	13.15±0.31	0.12±0.00
Deschampsia cespitosa	0.30±0.02	22.3±1.7	12.20	0.53±0.15	29701±1398	7.11±0.44	0.18±0.01
Dactylis glomerata	0.26±0.01	35.3±2.0	14.70	0.31±0.13	33118±1732	7.20±0.28	0.16±0.01
Festuca rubra	0.33±0.01	17.4±1.0	13.10	0.35±0.08	24133±982	7.09±0.13	0.16±0.00
Filipendula ulmaria	0.22±0.02	45.8±3.4	24.30	0.44±0.12	4863±162	6.43±0.39	0.35±0.04
Geranium sylvaticum	0.25±0.01	40.1±0.9	22.80	0.30±0.02	3782±799	8.61±1.36	0.44±0.09
Holcus lanatus	0.26±0.01	29.9±2.6	7.50	0.18±0.01	33940±1705	6.96±0.19	0.16±0.00
Hypochaeris radicata	0.14±0.01	23.4±1.5	13.50	0.09±0.00	23892±1221	7.83±0.23	0.12±0.00
Leontodon hispidus	0.12±0.01	32.0±1.3	24.20	0.12±0.01	19123±1513	12.44±0.47	0.13±0.01
Lolium perenne	0.21±0.01	35.4±2.3	14.00	0.22±0.03	30806±1309	8.49±0.27	0.24±0.08
Leucanthemum vulgare	0.15±0.01	22.8±0.5	19.10	0.15±0.01	18553±484	10.99±0.21	0.12±0.00
Phleum pratense	0.30±0.02	29.0±3.3	13.10	0.22±0.03	32446±2202	7.38±0.29	0.16±0.01
Plantago lanceolata	0.19±0.01	19.7±1.2	14.20	0.19±0.03	16260±732	10.37±0.34	0.15±0.00
Poa pratensis	0.35±0.03	19.9±2.9	12.90	0.44±0.09	17802±1821	6.73±0.59	0.23±0.01
Poa trivialis	0.26±0.01	46.7±2.7	13.50	0.31±0.12	34781±1218	8.35±0.18	0.16±0.01
Prunella vulgaris	0.18±0.01	25.8±1.7	19.20	0.11±0.01	19735±762	9.92±0.29	0.12±0.00
Ranunculus acris	0.18±0.01	28.6±1.2	18.30	0.19±0.02	8105±539	6.05±0.34	0.22±0.01
Rumex acetosa	0.10±0.01	40.4±2.3	21.00	0.42±0.03	12729±2849	6.95±0.78	0.32±0.08
Trisetum flavescens	0.28±0.02	40.9±2.9	18.00	0.35±0.06	29717±738	7.73±0.44	0.17±0.02

LDMC, leaf dry matter content; SLA, specific leaf area; Leaf N, leaf N content; RDMC, root dry matter content; SRL, specific root length; Root N, root N content; RTD, root tissue density.

Table S2 Minimum, maximum, and mean values for plant community attributes in our experiment.

	Minimum	Maximum	Average
Aboveground biomass (kg ha^{-1})	557	1492	955
Root biomass (kg ha^{-1})	966	2737	1867
Herb proportion	0	0.95	0.41
Functional diversity	0	105.8	40.1
Functional divergence	0	0.98	0.65
Functional richness	0	46.59	6.71
Rao's quadratic entropy	0	244.1	51.7
Evenness	0.05	0.99	0.71
Shannon's diversity	0	2.36	0.88
CWM SLA (mm^2 g^{-1})	18.1	41.9	29.1
CWM LDMC (g g^{-1})	0.14	0.33	0.23
CWM leaf N (mg g^{-1})	7.56	20.89	14.59
CWM SRL (cm g^{-1})	16863	34569	26132
CWM RDMC (g g^{-1})	0.10	0.43	0.24
CWM root N (mg g^{-1})	6.35	10.89	7.79
CWM RTD (g cm^{-3})	0.12	0.29	0.17

CWM, community weighted mean; LDMC, leaf dry matter content; SLA, specific leaf area; leaf N, leaf N content; RDMC, root dry matter content; SRL, specific root length; root N, root N content; RTD, root tissue density.

Table S3 Model selection procedure and statistics for the structural equation model (SEM) explaining ^{15}N pools and leaching (see Figs 1 and 6), only including leaf traits.

	Regressions deleted	df	AIC	dAIC	Chi-square	Chi-square difference	*P*-value
A-priori model		3	2873.6		1.10		
Model 2	Micr15N~root<br>Micr15N~herb<br>MicrCN~herb<br>Plant15N~SLA<br>Plant15N~rich<br>Plant15N~micr15N<br>Leach15N~micrCN<br>Leach15N~SLA<br>Leach15N~root<br>Leach15N~micr15N<br>Leach15N~herb<br>Leach15N~rich<br>SLA~~root<br>Root~rich<br>Root~Herb	10	2519.5	354.1	6.00	4.91	0.671
Model 3	Micr15N~SLA	11	2520.4	-0.9	8.87	2.86	0.091
Model 4	SLA~Herb	10	2515.2	5.2	7.69	1.18	0.278
Model 5	MicrCN~root	11	2516.5	-1.3	11.01	3.33	0.068

Table S4 The effect on *R*-squared of the removal of individual parameters from regressions containing multiple predictors in the final SEM for ^{15}N pools and leaching (Fig. 6), only including leaf traits.

Regression	Removal of:	Reduction in *R*-squared:
Micr15N~MicrCN+SLA	MicrCN<br>SLA	0.322<br>0.037
Plant15N~micrCN+herb+root+SLA	micrCN<br>herb<br>root<br>SLA	0.029<br>0.074<br>0.527<br>0.006

Table S5 Model selection procedure and statistics for the structural equation model (SEM) explaining ^{15}N pools and leaching (see Figs 1 and 7), including leaf traits as well as root traits.

	Regressions deleted	df	AIC	dAIC	Chi-square	Chi-square difference	*P*-value
A-priori model		5	3233.8		1.17		
Model 2	Micr15N~SLA<br>Micr15N~herb<br>MicrCN~herb<br>MicrCN~SLA<br>Plant15N~SLA<br>Plant15N~rich<br>Leach15N~micrCN<br>Leach15N~SLA<br>Leach15N~root<br>Leach15N~micr15N<br>Leach15N~herb<br>Leach15N~rich<br>SLA~herb<br>RTD~herb<br>Root~herb<br>SLA~~RTD<br>RTD~~root<br>SLA~~root<br>Root~rich	6	2546.5	687.3	1.37	0.20	0.656
Model 3	Micr15N~root<br>Plant15N~micrCN<br>Leach15N~RTD	9	2546.4	0.1	7.22	5.86	0.119

Table S6 The effect on *R*-squared of the removal of individual parameters from regressions containing multiple predictors in the final SEM for ^{15}N pools and leaching (Fig. 7), including leaf and root traits.

Regression	Removal of:	Reduction in *R*-squared:
Micr15N~micrCN+RTD(+root)	micrCN	0.368
	RTD	0.069
	root	0.022
MicrCN~RTD+root	RTD	0.013
	root	0.073
Plant15N~RTD+herb+root	RTD	0.067
	Herb	0.075
	root	0.483

Table S7 Model selection procedure and statistics for the structural equation model (SEM) explaining ^{15}N retention (see Figs 2 and 8), including leaf traits as well as root traits.

	Regressions deleted	df	AIC	dAIC	Chi-square	Chi-square difference	*P*-value
A-priori model		2	2285.8		0.33		
Model 2	RootN~rich<br>Nroot~rootN<br>Nroot~herb<br>Nroot~rich<br>Root~herb<br>Ret~hebr<br>Ret~root<br>Ret~rootN<br>Ret~LDMC<br>DMC~~rootN<br>Herb~~rich<br>DMC~~root	12	2266.9		7.45	7.11	0.715
Model 3	Root~rich	7	1936.9		6.90	0.54	0.99
Model 4	RootN~herb<br>RootN~root	5	1552.0		4.30	2.60	0.273

Table S8 The effect on *R*-squared of the removal of individual parameters from regressions containing multiple predictors in the final SEM for ^{15}N retention (Fig. 8).

Regression	Removal of:	Reduction in *R*-squared:
Nroot~root+LDMC+herb	root	0.605
	LDMC	0.035
	herb	0.001

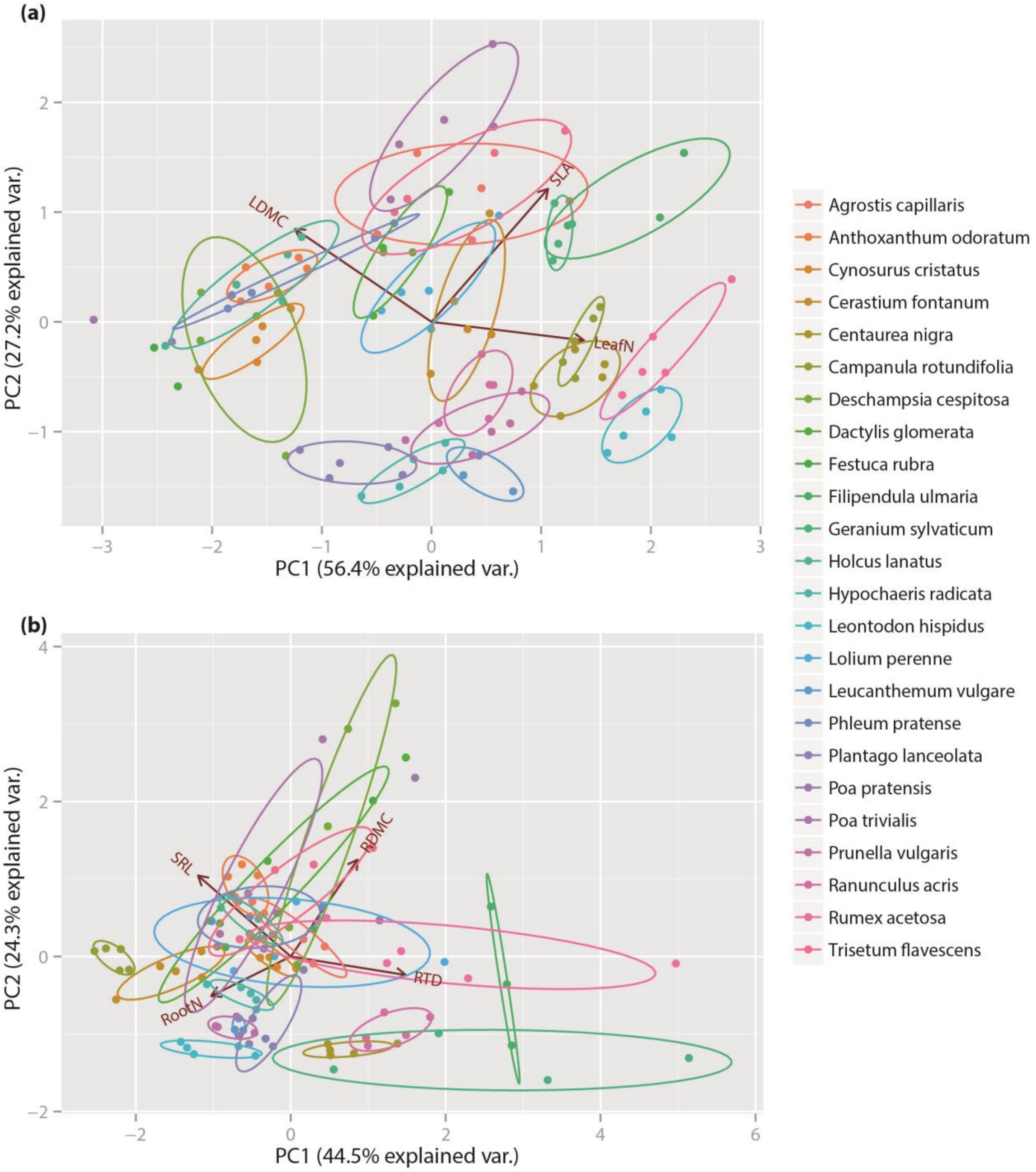

Fig. S1 PCA biplots for leaf traits (a) and root traits (b) for the 24 species used in the experiment. Points show individual plants; ellipses show normal contour lines (probability of 68%) for species. LDMC, leaf dry matter content; SLA, specific leaf area; leaf N, leaf N content; RDMC, root dry matter content; SRL, specific root length; root N, root N content; RTD, root tissue density.

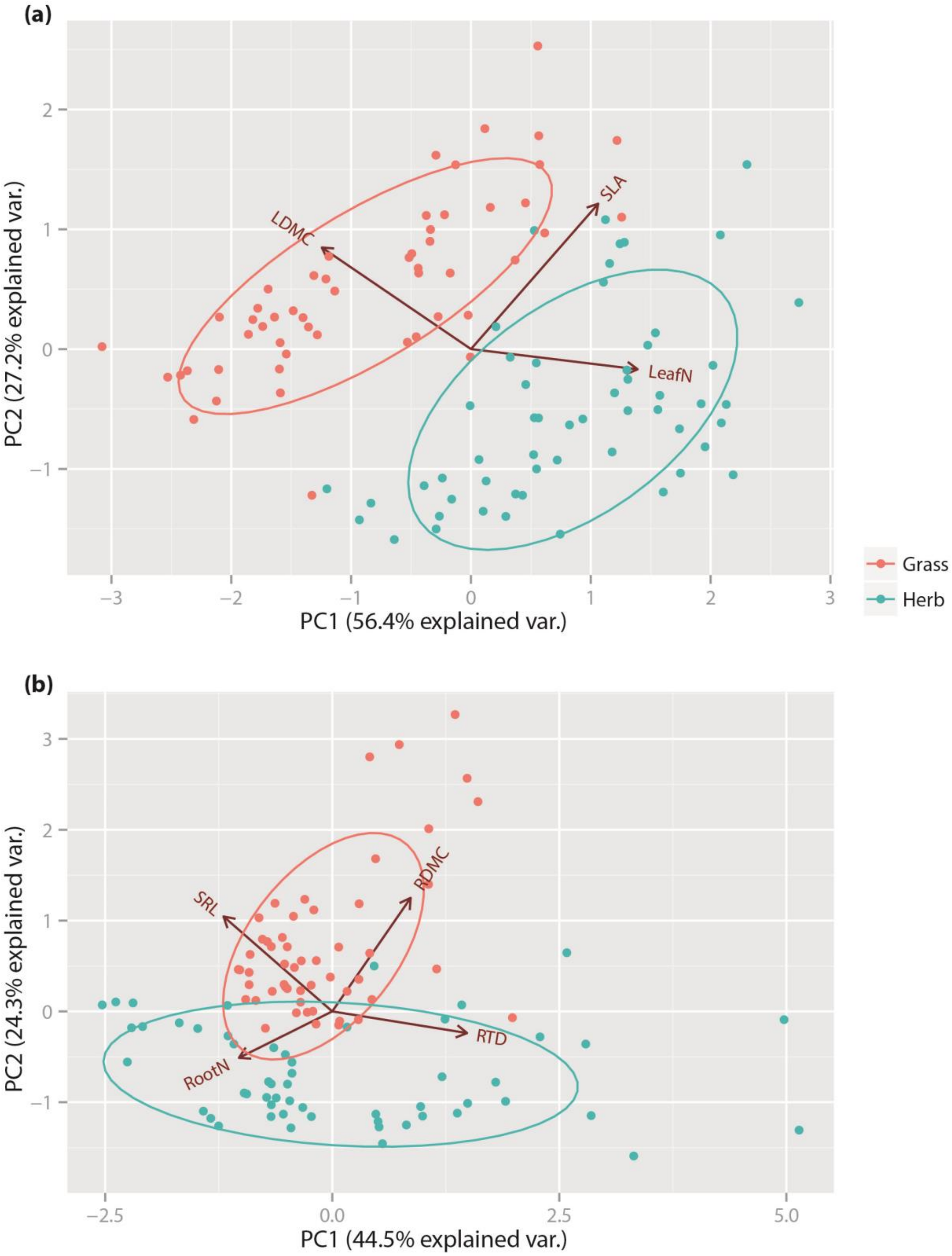

Fig. S2 PCA biplots for leaf traits (a) and root traits (b) for the 24 species used in the experiment. Points show individual plants; ellipses show normal contour lines (probability of 68%) for grasses and herbs. LDMC, leaf dry matter content; SLA, specific leaf area; leaf N, leaf N content; RDMC, root dry matter content; SRL, specific root length; root N, root N content; RTD, root tissue density.

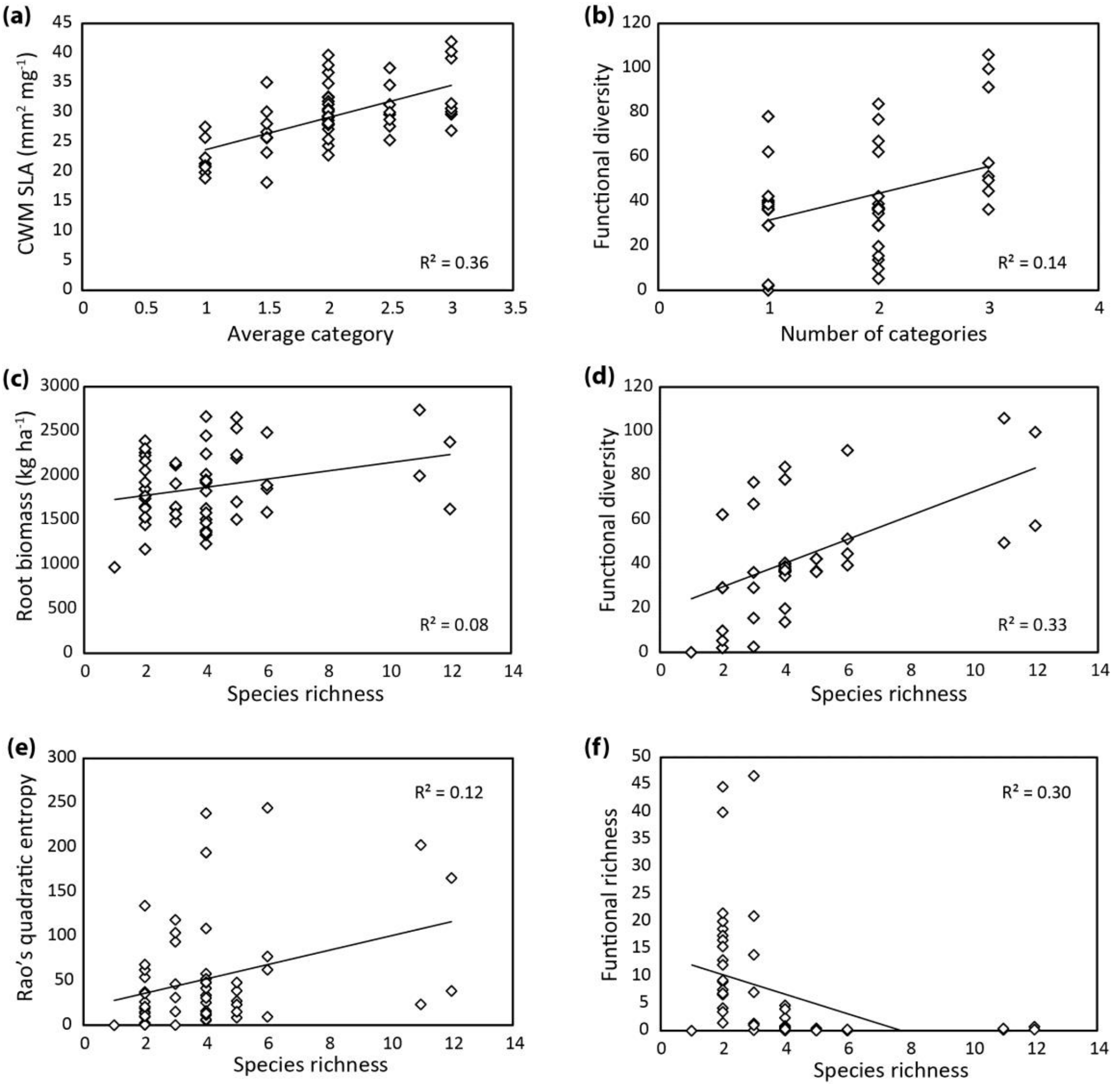

Fig. S3 Treatment (average trait category, number of trait categories, and species richness, see Tables 1 and 2) effects on plant community attributes and ^{15}N pools: (a) community weighted mean (CWM) specific leaf area (SLA) increased with category rank, (b) functional diversity increased with number of trait categories, (c) root biomass increased with realised species richness, (d) functional diversity increased with realised species richness, (e) Rao's quadratic entropy increased with species richness, (f) functional richness decreased with species richness. Symbols represent individual observations. See text and Table 5 for statistics.

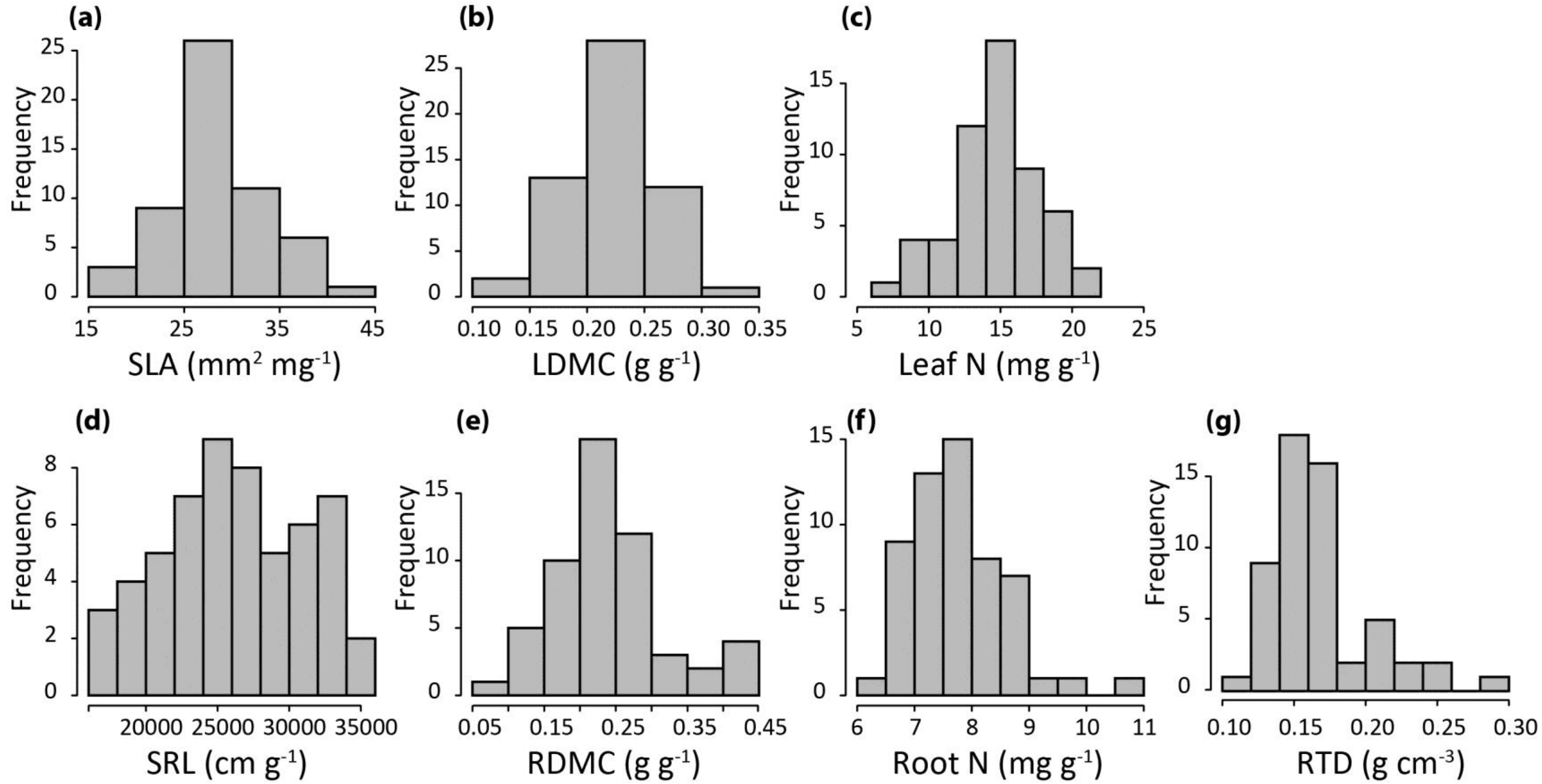

Fig. S4 Histograms showing frequency distributions for community weighted mean (CWM) leaf (a–c) and root traits (d–g) for the experimental communities. SLA, specific leaf area; LDMC, leaf dry matter content; leaf N, leaf N content; RDMC, root dry matter content; SRL, specific root length; root N, root N content; RTD, root tissue density.

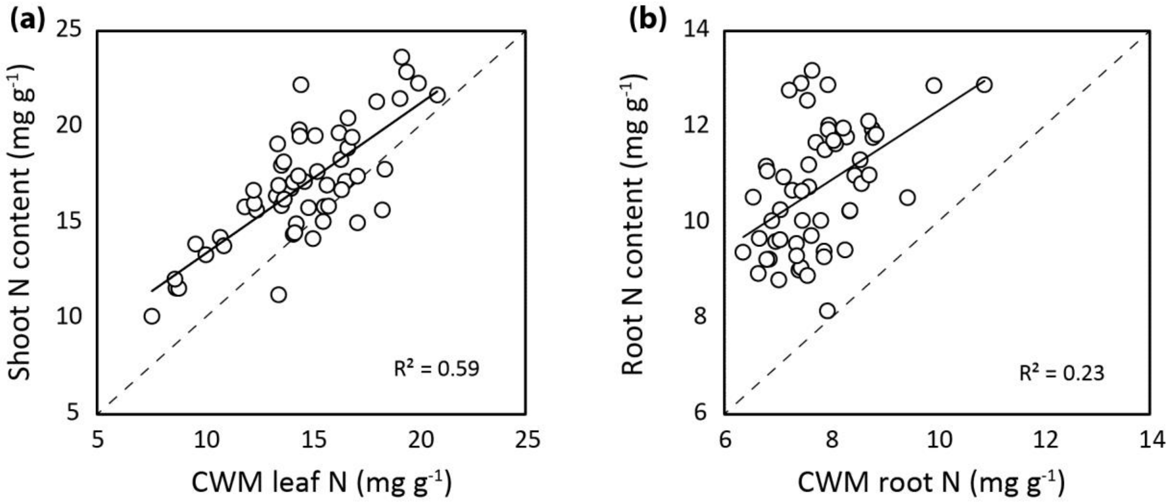

Fig. S5 The relationship between community weighted mean (CWM) leaf N and root N content calculated from individual abundances and species averaged traits reported in Table S1, and measured total community shoot and root N content.

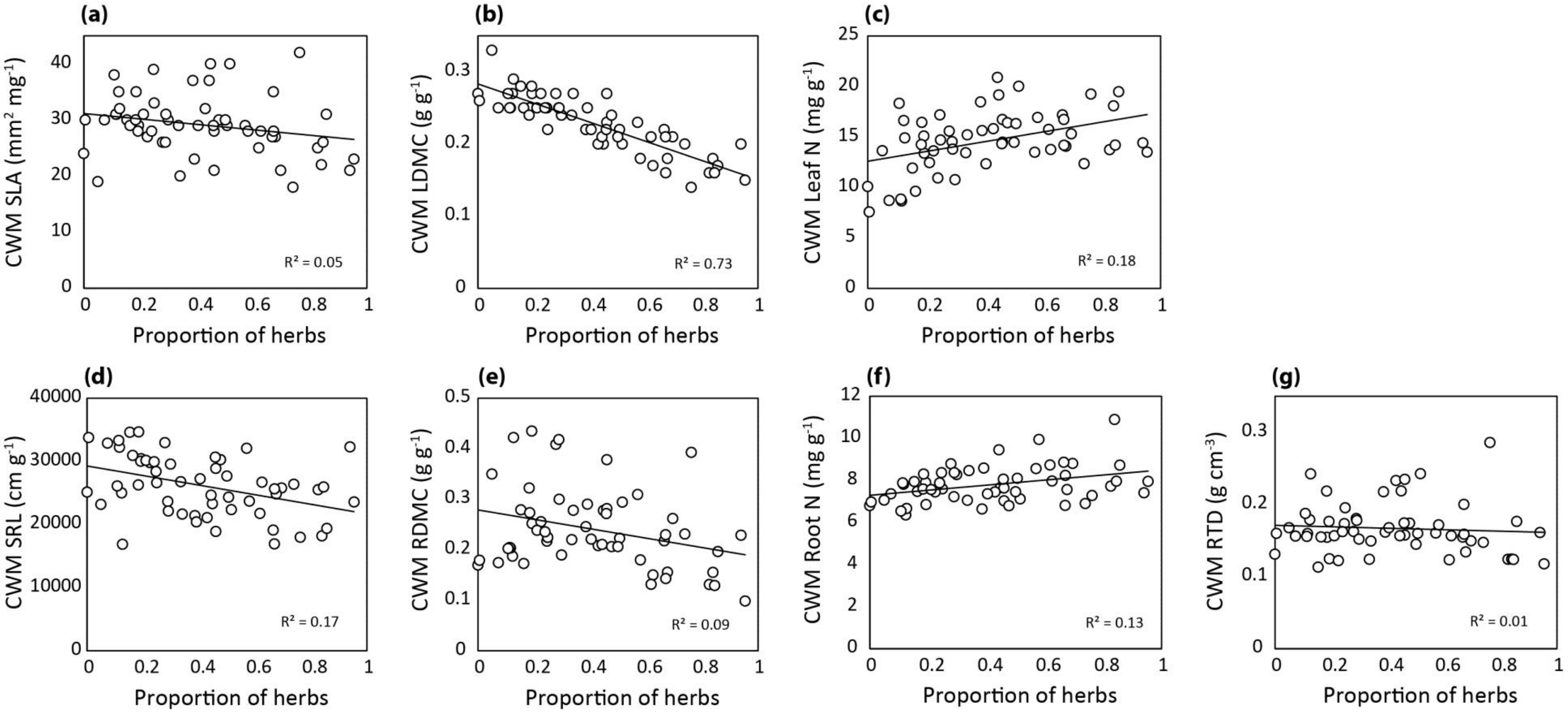

Fig. S6 The effect of the proportion of herb biomass of total community biomass on values for CWM traits, for leaf traits (a–c) and root traits (d–g). CWM LDMC (b, $P < 0.001$), CWM Leaf N (c, $P = 0.001$), CWM SRL (d, $P = 0.002$), and CWM Root N (f, $P < 0.001$) were significantly affected by the proportion of herbs. CWM, community weighted mean; SLA, specific leaf area; LDMC, leaf dry matter content; leaf N, leaf N content; RDMC, root dry matter content; SRL, specific root length; root N, root N content; RTD, root tissue density.

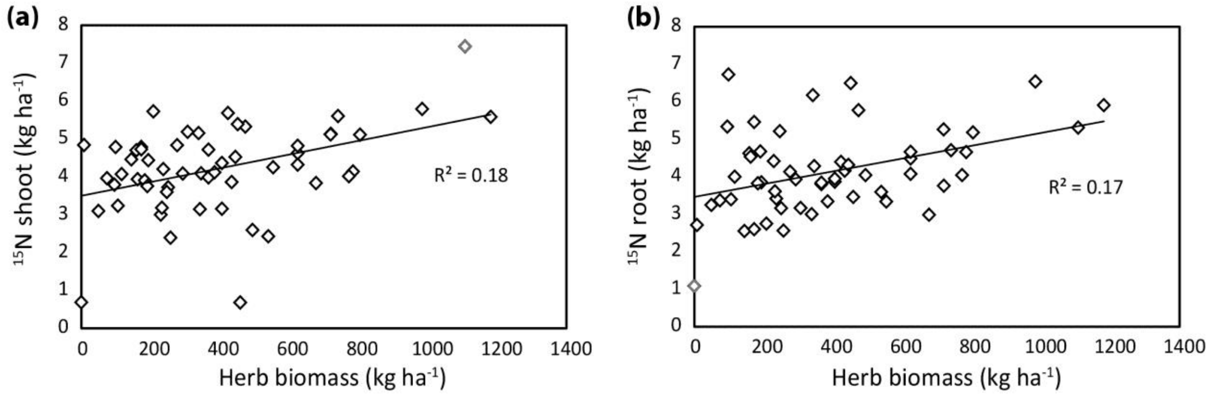

Fig. S7 Relationships between aboveground ^{15}N uptake and herb biomass. ^{15}N uptake of both shoots (a) and roots (b) was higher with increased herb biomass. See main text for statistics.

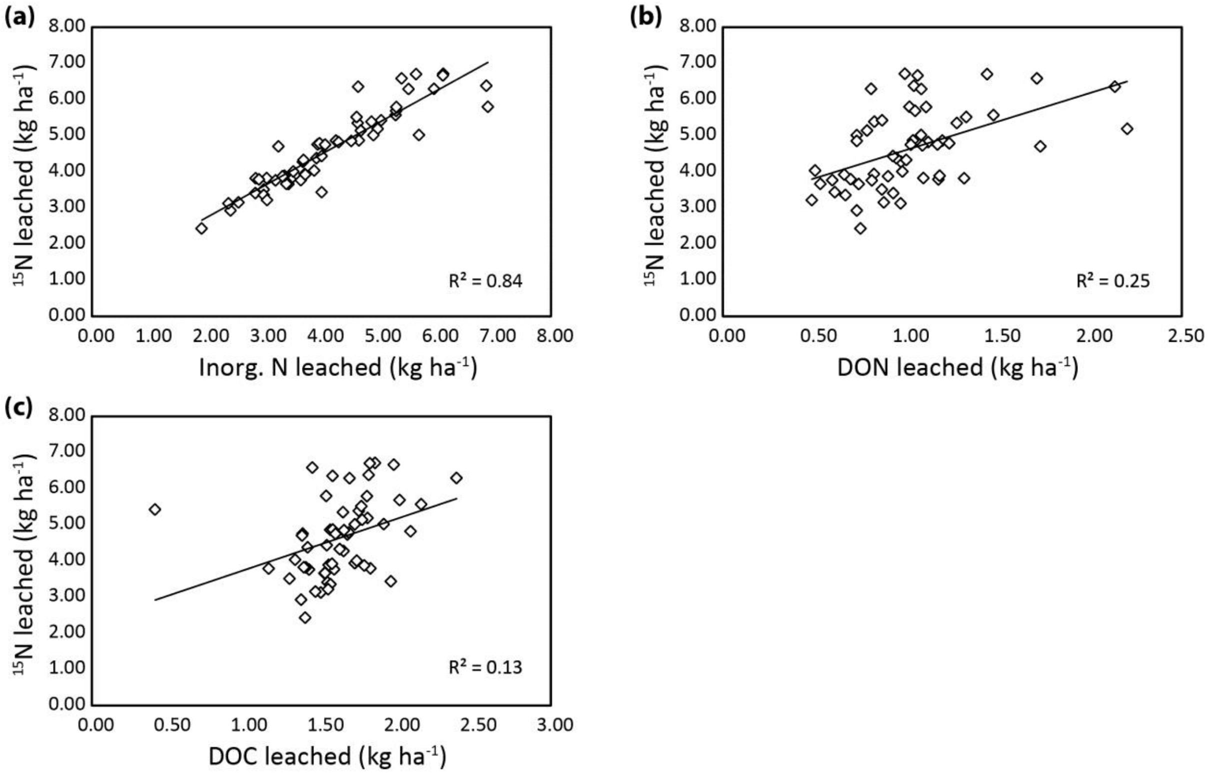

Fig. S8 Relationship between ^{15}N leached and the amounts of inorganic N (a), dissolved organic N (DON) (b), and dissolved organic C (DOC) leached (c). See main text for statistics.

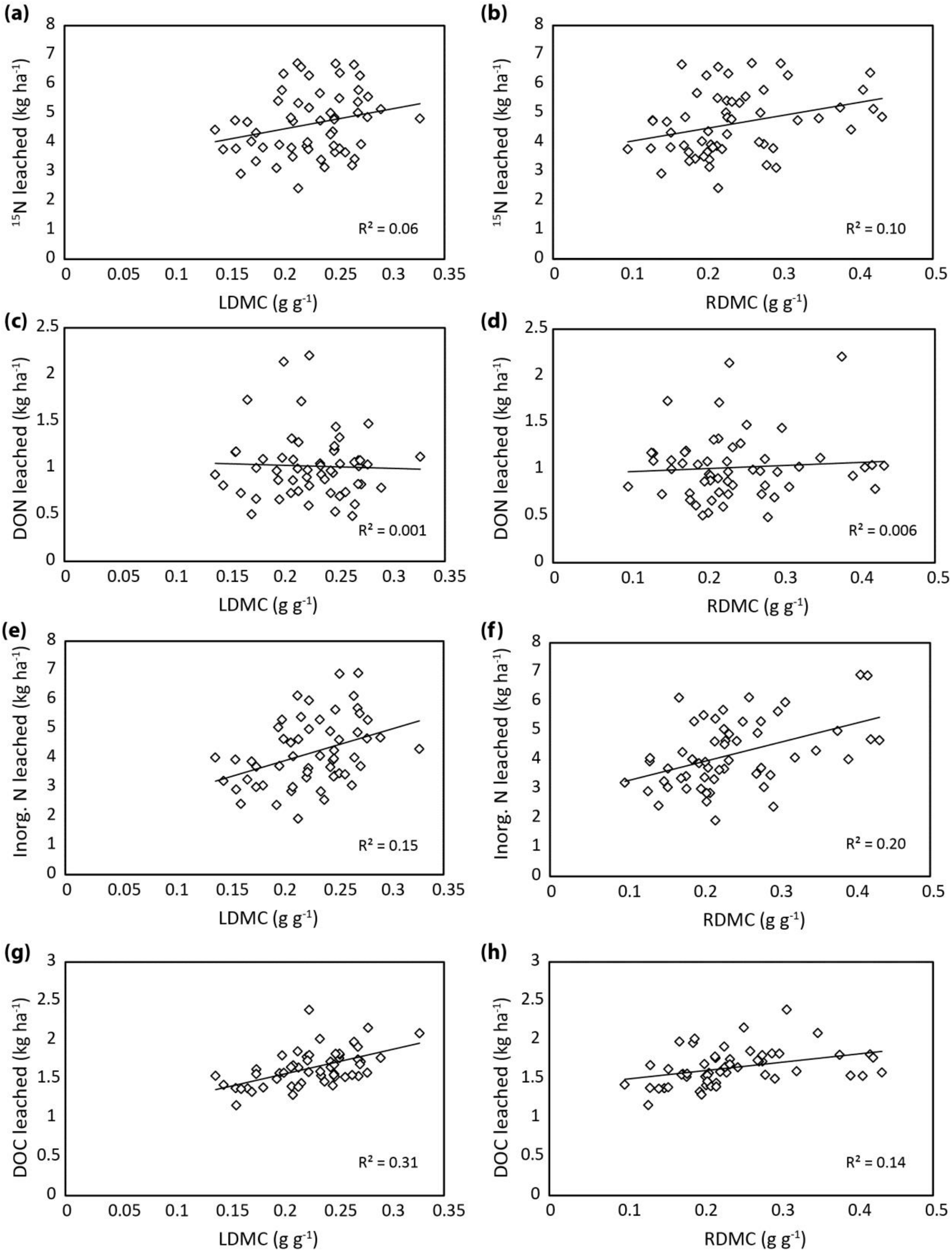

Fig. S9 Amounts of ^{15}N (a, b; P = 0.069 and P = 0.016), DON (c, d; P = 0.798 and P = 0.577), inorganic N (e, f; P = 0.004 and P < 0.001), and DOC (g, h; P < 0.001 and P = 0.005) leached as explained by leaf dry matter content (LDMC) and root dry matter content (RDMC), respectively.

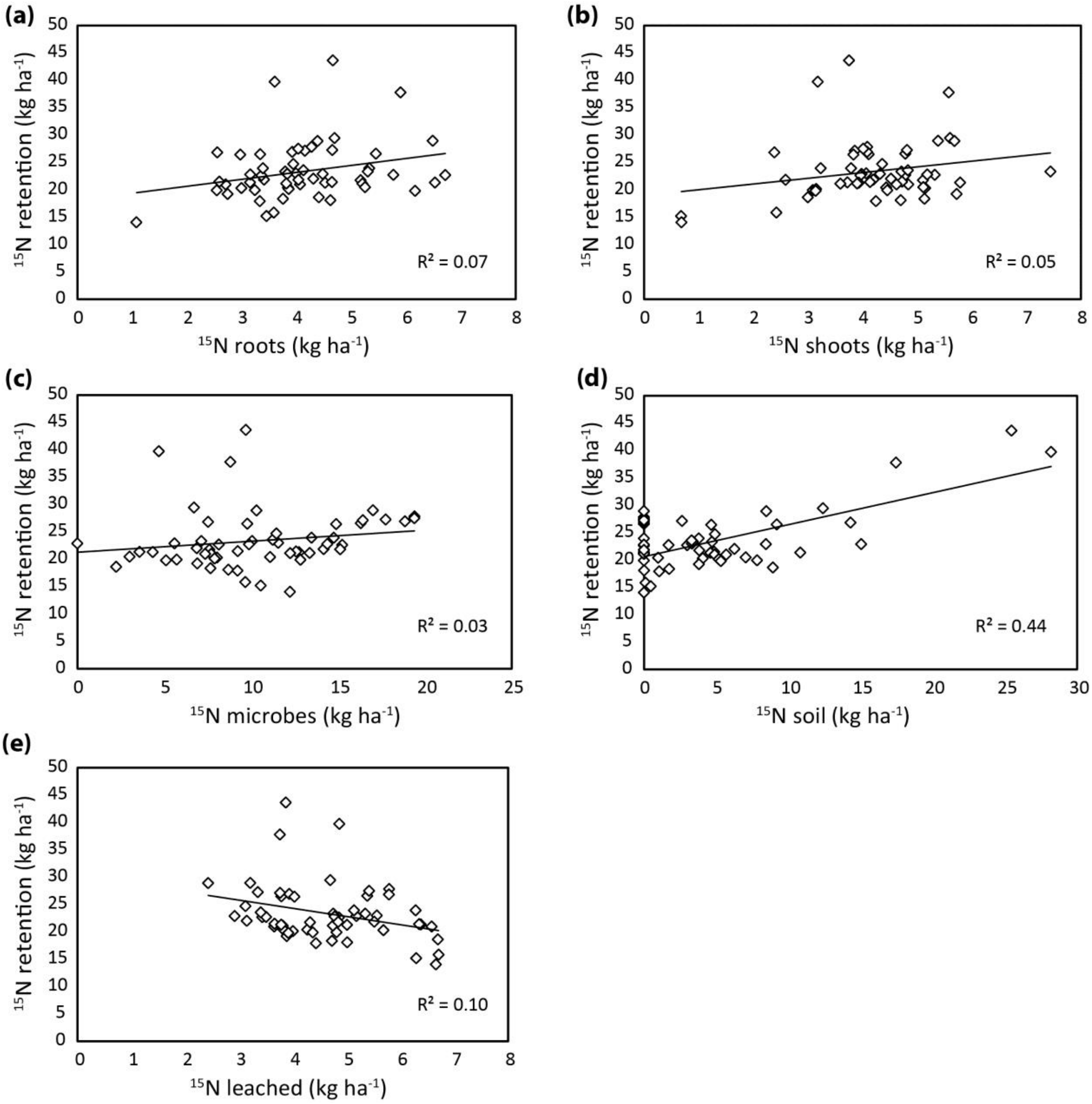

Fig. S10 Relationships between individual ^{15}N pools and the amount of ^{15}N retained in the system (the sum of plant, soil, and microbial ^{15}N).
